# Protein kinase G2 activation restores Wnt signaling and bone mass in glucocorticoid-induced osteoporosis in mice

**DOI:** 10.1172/jci.insight.175089

**Published:** 2024-06-17

**Authors:** Shyamsundar Pal China, Hema Kalyanaraman, Shunhui Zhuang, Justin A. Cabriales, Robert L. Sah, Renate B. Pilz

**Affiliations:** 1 Department of Medicine and; 2 Department of Bioengineering, UCSD, La Jolla, California, USA.

**Keywords:** Bone biology, Cyclic nucleotides, Osteoclast/osteoblast biology, Osteoporosis

## Abstract

Osteoporotic fractures are a major complication of long-term glucocorticoid therapy. Glucocorticoids transiently increase bone resorption, but they predominantly inhibit bone formation and induce osteocyte apoptosis, leading to bone loss. Current treatments of glucocorticoid-induced osteoporosis aim mainly at reducing bone resorption and are, therefore, inadequate. We previously showed that signaling via the NO/cGMP/protein kinase G pathway plays a key role in skeletal homeostasis. Here, we show that pharmacological PKG activation with the guanylyl cyclase-1 activator cinaciguat or expression of a constitutively active, mutant PKG2^R242Q^ restored proliferation, differentiation, and survival of primary mouse osteoblasts exposed to dexamethasone. Cinaciguat treatment of WT mice or osteoblast-specific expression of PKG2^R242Q^ in transgenic mice prevented dexamethasone-induced loss of cortical bone mass and strength. These effects of cinaciguat and PKG2^R242Q^ expression were due to preserved bone formation parameters and osteocyte survival. The basis for PKG2’s effects appeared to be through recovery of Wnt/β-catenin signaling, which was suppressed by glucocorticoids but critical for proliferation, differentiation, and survival of osteoblast-lineage cells. Cinaciguat reduced dexamethasone activation of osteoclasts, but this did not occur in the PKG2^R242Q^ transgenic mice, suggesting a minor role in osteoprotection. We propose that existing PKG-targeting drugs could represent a novel therapeutic approach to prevent glucocorticoid-induced osteoporosis.

## Abstract 2

Restoration of Wnt signaling by treatment with a cGMP-elevating agent or osteoblast-specific expression of a constitutively active PKG2 prevents glucocorticoid-induced osteoporosis in mouse models.

## Graphical abstract

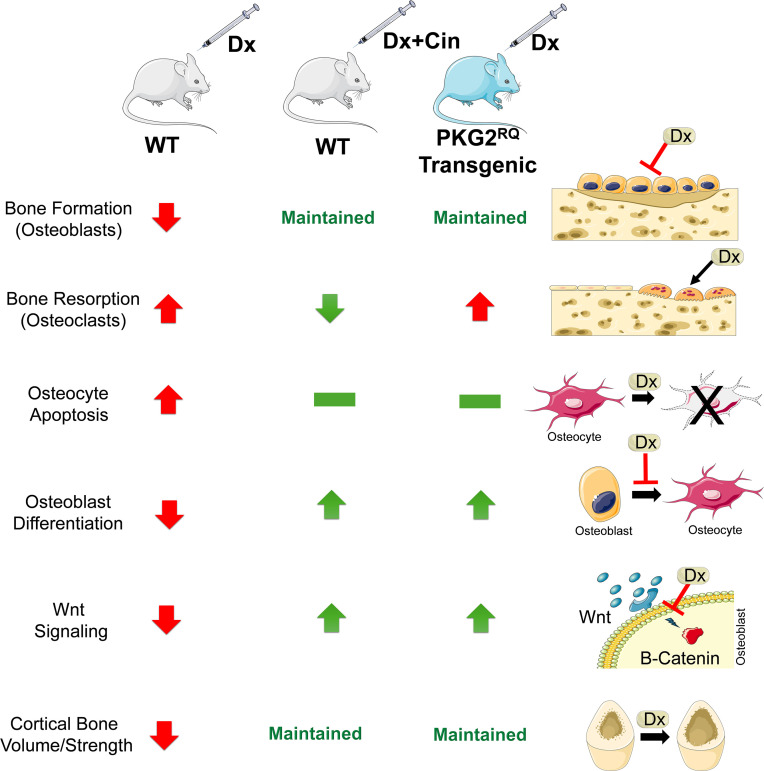

## Introduction

The balance between bone formation and resorption maintains adult bone mass; inadequate formation or excess resorption of bone leads to osteoporosis, characterized by low bone mass and increased fracture risk. Glucocorticoid-induced osteoporosis (GIOP) is the most common form of drug-induced osteoporosis, with ~1% of U.S. adults and 3% of those older than 50 years prescribed glucocorticoids for autoimmune and inflammatory disorders or malignancies (1). Almost one-third of glucocorticoid-treated patients experience an osteoporotic fracture; fracture risk increases with age, dose, and duration of treatment (2). Thus, patients receiving prednisone at > 10 mg per day for more than 3 months have about a 7- and 17-fold increased risk of hip and vertebral fractures, respectively, compared with age-matched controls (3). Even though GIOP-related fractures are a major cause of disability, economic stress, and even death, GIOP remains severely underrecognized and undertreated, especially in males and younger adults (4).

Current guidelines for the prevention and treatment of GIOP are based on clinical fracture risk assessment and include calcium and vitamin D supplementation, but pharmacological therapies remain unsatisfactory (1, 4, 5). Antiresorptive agents such as bisphosphonates and denosumab, a neutralizing antibody against receptor of activated NF-κB ligand (RANKL), are typically used as a first-line treatment, but they reduce bone formation (1, 4, 5). Anabolic agents such as parathyroid hormone analogs enhance bone formation, but they have limitations and are only conditionally recommended, due to a paucity of fracture prevention data (5, 6). Thus, while GIOP-induced fractures may be preventable, new and more effective treatment options are urgently needed.

The initial phase of GIOP-induced bone loss is characterized by a rapid suppression of bone formation and a transient increase in bone resorption; the latter is because of increased osteoclast number and activity, driven by changes in osteoclast differentiation factors, such as RANKL and osteoprotegerin (OPG), and increased osteoclast survival (2, 4). Glucocorticoids suppress bone formation long-term, leading to the low bone turnover state typical for GIOP in humans and mice (2, 4, 7). Reduced bone formation persists due to a shift in mesenchymal stem cell differentiation from osteogenesis to adipogenesis and a profound reduction in osteoblast maturation, function, and survival (2, 4, 7). Glucocorticoids also reduce bone quality by inducing osteocyte apoptosis and altering extracellular matrix composition and water content (2, 4, 7). Many of these glucocorticoid effects on osteoblast lineage cells are mediated by alterations in Wnt/β-catenin signaling, with glucocorticoids decreasing the expression of Wnt ligands, such as Wnt-16 and Wnt-10b, and increasing Wnt inhibitors, such as dickkopf-1 (*DKK1*) and sclerostin (*SOST*) (8–12)

Nitric oxide (NO) plays a central role in skeletal homeostasis, acting as a second messenger in mechanical and hormonal stimulation of bone formation (13). NO enhances proliferation, differentiation, and survival of cells in the osteoblast lineage via activation of guanylyl cyclase-1, generation of cGMP, and activation of type 1 and 2 protein kinase G (PKG1 and -2) (13). We previously showed that pharmacological PKG activation, or osteoblast-specific expression of a partly activated PKG2 (PKG2^R242Q^), protects mice against osteoporosis caused by type 1 diabetes by improving bone formation and preventing osteocyte apoptosis (14, 15). Based on the bone-anabolic effects of PKG2, we hypothesized that pharmacological or genetic PKG2 activation may prevent GIOP, and we tested this hypothesis in a dexamethasone-induced (Dx-induced) osteoporosis model in male C57BL/6NHsd mice. We found that cinaciguat, a guanylyl cyclase-1 activating agent that activates PKG1 and -2, prevented GIOP in WT mice. Consistent with these effects being mediated by PKG2, Col1a1-*Prkg2^R242Q^* transgenic mice were largely resistant to dexamethasone-induced bone loss. PKG activation restored osteoblast functions and Wnt/β-catenin signaling after dexamethasone exposure both in vitro (in primary osteoblasts) and in vivo (in mice).

## Results

### Cinaciguat prevents dexamethasone-induced inhibition of osteoblast proliferation, survival, and differentiation.

Based on our earlier work showing that NO/cGMP/PKG signaling enhances proliferation, survival, and differentiation of osteoblast-lineage cells (13, 14, 16, 17), we hypothesized that PKG activation with the NO-independent guanylyl cyclase-1 activator cinaciguat could protect osteoblasts from the detrimental effects of dexamethasone, a glucocorticoid with almost no mineralocorticoid activity (4). We previously showed that guanylyl cyclase activation by cinaciguat increases intracellular cGMP concentrations and activates PKG1 and -2 in osteoblasts (16).

**Figure 1:**
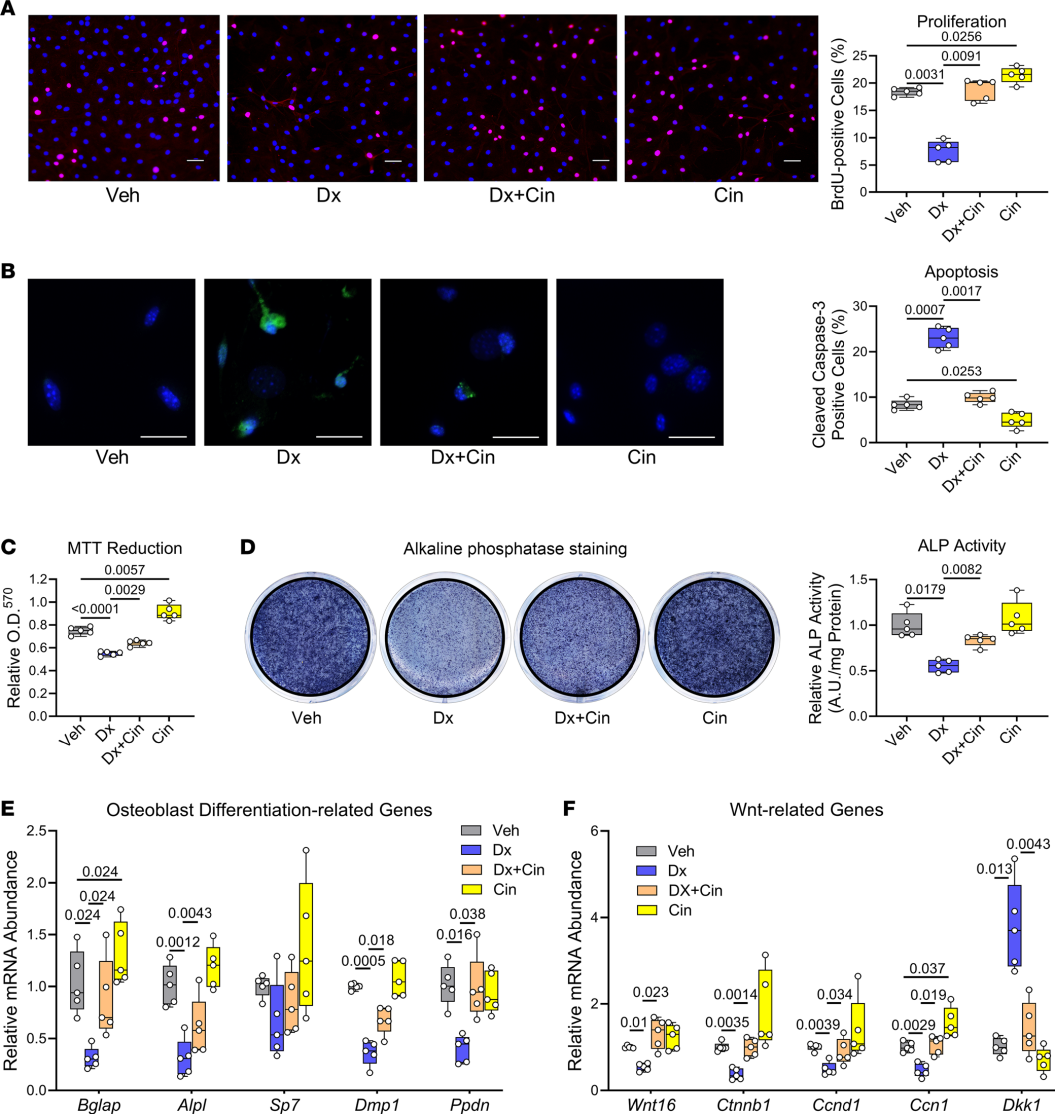
Cinaciguat prevents dexamethasone-induced inhibition of osteoblast proliferation, survival, and differentiation. Primary osteoblasts isolated from WT C57BL/6Hsd mice were treated for the indicated time with vehicle (Veh, 0.1% DMSO, gray bars), dexamethasone (Dx, blue bars), dexamethasone + cinaciguat (Dx+Cin, orange bars), or cinaciguat alone (Cin, yellow bars) in medium containing 1% FBS, unless indicated otherwise. (**A**) Proliferation was assessed by BrdU uptake into S-phase nuclei, identified by immunofluorescence staining; cells were incubated for 24 hours in medium containing BrdU in the absence or presence of the drugs. Scale bar: 50 μm. (**B**) Apoptosis was assessed by immunostaining for cleaved caspase-3 after incubating cells for 18 hours in the absence or presence of the drugs. Sclae bar: 25 μm. (**C**) Metabolic activity was assessed by MTT reduction to formazan; cells were incubated for 48 hours in the absence or presence of the drugs, with MTT added for the last 4 hours. (**D**) Alkaline phosphatase (ALP) activity was assessed by staining with p-nitrophenolphosphate, after 7 days of culture of postconfluent cells in osteoblast differentiation medium containing 10% FBS, ascorbate, and β-glycerolphosphate, in the absence or presence of Dx and/or Cin. Activity was normalized to protein concentration, and the mean of vehicle-treated cells was assigned a value of 1. (**E** and **F**) Relative mRNA abundance was quantified by RT-qPCR after culture for 7 days in differentiation medium (**E**) or after 24 hours in growth medium (**F**); mRNA expression was normalized to the housekeeping gene hypoxanthine phosphoribosyltransferease (*Hprt)*, and the mean ΔCt observed in WT cells was assigned a value of 1. Osteoblast-differentiation–related genes (**E**): *Bglap*, osteocalcin; *Alpl*, alkaline phosphatase; *Sp7*, osterix; *Dmp1*, dentin matrix acidic phophoprotein-1; *Ppdn*, podoplanin/GP38. Wnt-related genes (**F**): *Wnt16*, Wnt family member 16; *Ctnnb1*, β-catenin; *Ccnd1*, cyclin D; *Ccn1*, cellular communication network factor-1; *Dkk1*, dickkopf-1. The box-and-whisker box plots show medians and 25th to 75th percentiles of 5 independent experiments; data were analyzed by repeated-measures 1-way ANOVA with Geisser-Greenhouse correction (not assuming equal variances), followed by Holm-Šidák’s multiple-comparison test.

Treating primary osteoblasts from male mice with dexamethasone inhibited cell proliferation by ~60% and almost tripled the number of apoptotic cells, but cinaciguat fully reversed these effects; by itself, cincaciguat increased proliferation and reduced apoptosis, as we have previously shown in female osteoblasts (16) (Figure 1, A and B). Consistent with increased apoptosis, dexamethasone — in a dose-dependent fashion — inhibited MTT reduction to formazan, with cinaciguat increasing MTT reduction in the presence and absence of dexamethasone (Figure 1C and Supplemental Figure 1A; supplemental material available online with this article; https://doi.org/10.1172/jci.insight.175089DS1).

**Figure 2:**
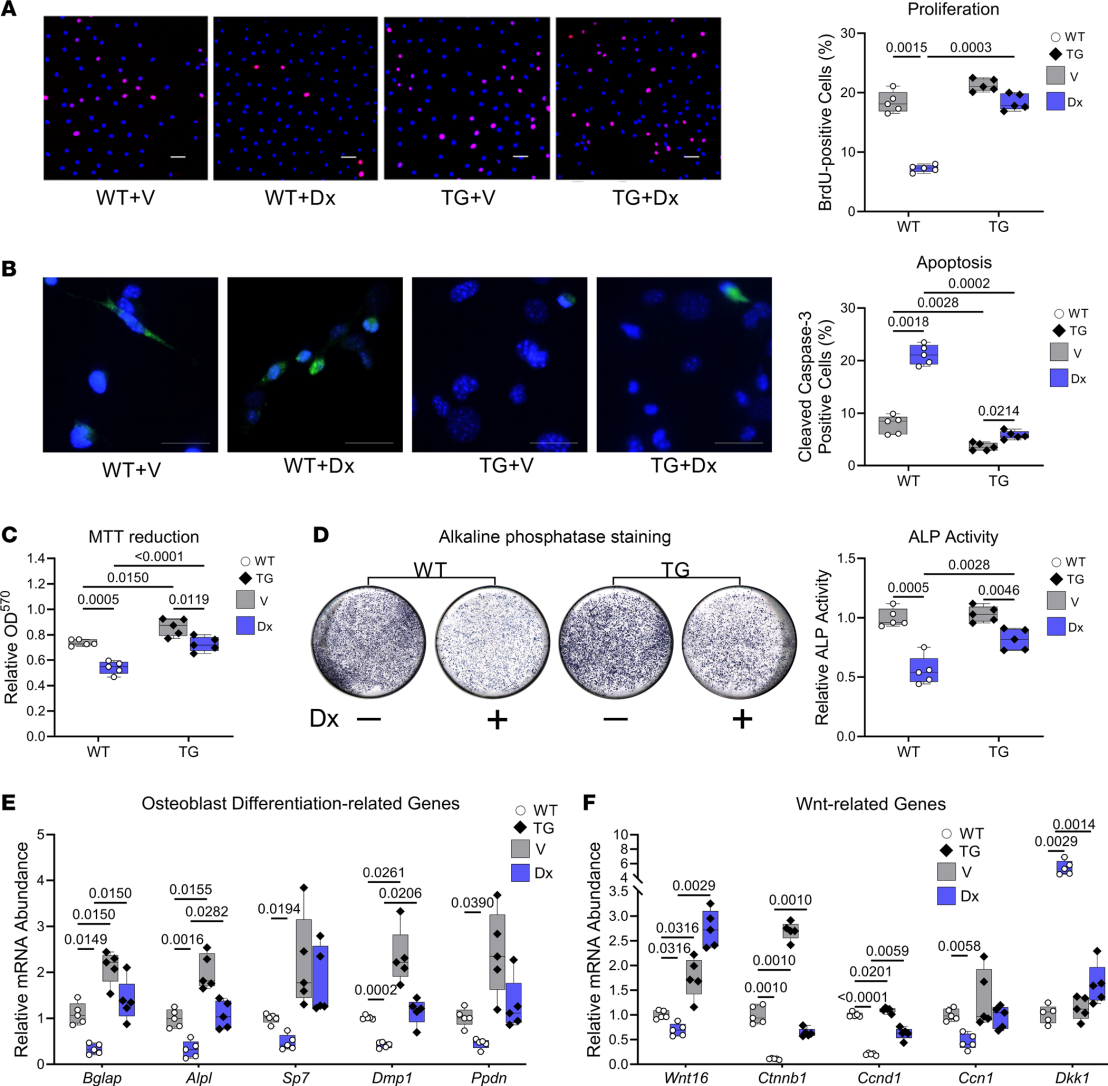
Primary osteoblasts expressing a partly activated PKG2 are protected against dexamethasone toxicity. Primary osteoblasts isolated from Col1a1-PKG2^R242Q^ transgenic mice (TG, closed symbols) and their WT littermates (WT, open symbols) were treated with vehicle (gray bars) or dexamethasone (Dx, blue bars) in medium containing 1% FBS, unless indicated otherwise. (**A**) Proliferation was assessed by BrdU uptake into S-phase nuclei as described in Figure 1A; cells were cultured with BrdU for 24 hours in the absence or presence of Dx. Scale bar: 50 μm. (**B**) Apoptosis was assessed by cleaved caspase-3 staining as in Figure 1B; cells were incubated for 18 hours in the absence or presence of Dx. Scale bar: 25 μm. (**C**) Metabolic activity was assessed by MTT reduction as described in Figure 1C; cells were incubated in the absence or presence of Dx for 48 hours. (**D**) Alkaline phosphatase activity was assessed as in Figure 1D; cells were cultured for 7 days in osteoblast differentiation medium with 10% FBS. (**E** and **F**) Relative mRNA abundance of the indicated genes was quantified and normalized as described in Figure 1E, after 7 days in differentiation medium (**E**) or after 24 hours in growth medium (**F**). Box-and-whisker box plots for 5 independent experiments as in Figure 1, but data were analyzed by 2-way ANOVA.

When postconfluent osteoblasts were cultured for 7 days in the presence of ascorbate and β-glycerolphosphate to induce differentiation, alkaline phosphatase activity and expression of *Alpl* and *Bglap* mRNA (encoding alkaline phosphatase and osteocalcin, respectively) were reduced by dexamethasone; these differentiation markers were largely restored by cinaciguat, which itself increased *Bglap* mRNA expression (Figure 1, D and E). Neither dexamethasone nor cinaciguat affected mRNA levels of the transcription factor osterix (*Sp7*) (Figure 1E). Osteocyte marker gene expression (*Dmp1* and *Ppdn*) was reduced in the presence of dexamethasone, but cinaciguat prevented this reduction, without having an effect in the absence of dexamethasone (Figure 1E). We used 0.5 μM dexamethasone in differentiating osteoblast cultures to minimize the drug’s proapoptotic effects in these long-term experiments; all other in vitro experiments in Figures 1 and 2 were performed with 2.5 μM dexamethasone.

In undifferentiated osteoblasts, dexamethasone suppressed mRNA expression of the Wnt ligand *Wnt16*, the Wnt-regulated transcription factor β-catenin (*Ctnnb1*), and β-catenin target genes (cyclin D [*Ccnd1*] and cellular communication network factor-1 [*Ccn1*]), while increasing expression of the Wnt inhibitor *Dkk1* (Figure 1F). Cinaciguat fully restored *Wnt16,* *Ctnnb1*, *Ccnd1*, and *Ccn1* expression and reversed the glucocorticoid effects on *Dkk1* (Figure 1F). In the absence of dexamethasone, cinaciguat increased *Ccn1* mRNA expression, and there was a trend toward increased *Ctnnb1* and *Ccnd1* expression (Figure 1F).

Thus, cinaciguat largely protected osteoblasts from the detrimental effects of dexamethasone, and this appeared to be accomplished at least partly by preserving Wnt/β-catenin signaling. Treating osteoblasts with a membrane-permeable cGMP analog mimicked the effects of cinaciguat, protecting cells from dexamethasone-induced apoptosis, and restoring MTT reduction and alkaline phosphatase activity in dexamethasone-treated cells (Supplemental Figure 1, B–D).

### Primary osteoblasts expressing a partly activated PKG2 are protected against dexamethasone toxicity.

If cinaciguat was acting via PKG activation to reverse dexamethasone effects in osteoblasts, then cells with increased PKG activity should be resistant to the deleterious effects of dexamethasone. To test this premise, we isolated primary osteoblasts from the bones of young adult male Col1a1*-Prkg2^R242Q^* transgenic mice and their WT littermates. Osteoblasts from the transgenic mice express a partly activated PKG2 (PKG2^R242Q^) and show a 2- to 3-fold increase in basal and cGMP-stimulated PKG2 activity compared with WT osteoblasts; PKG1 activity is unchanged (15).

The PKG2^R242Q^-expressing osteoblasts showed a trend toward higher BrdU incorporation (*P* = 0.051) and significantly reduced cleaved caspase-3 staining compared with WT osteoblasts under basal conditions (Figure 2, A and B). Dexamethasone suppressed proliferation and increased apoptosis in the WT cells, but the PKG2^R242Q^-expressing cells were highly resistant to dexamethasone (Figure 2, A and B). Dexamethasone decreased MTT reduction in both WT and PKG2^R242Q^-expressing cells, but the latter cells were less sensitive to dexamethasone at all concentrations tested and had higher basal MTT reduction than WT cells (Figure 2C and Supplemental Figure 1E). Alkaline phosphatase activity was similar in differentiated WT and PKG2^R242Q^-expressing cells, but it was reduced less by dexamethasone in PKG2^R242Q^-expressing cells compared with WT cells (Figure 2D). Osteoblasts expressing PKG2^R242Q^ were completely protected from the dexamethasone-induced decrease in *Bglap* and *Wnt16* mRNA, largely protected from the dexamethasone-induced increase in *Dkk1*, and partly protected from dexamethasone-induced reductions in *Alpl,* *Dmp1, Ppdn*, *Ctnnb1,* and *Ccnd1* mRNA (Figure 2, E and F). *Bglap,* *Alpl,* *Dmp1*, *Wnt16*, *Ctnnb1*, and *Ccnd1* mRNA abundance was higher in both untreated and dexamethasone-treated PKG2^R242Q^-expressing cells compared with WT cells (Figure 2, E and F). *Alpl* mRNA and alkaline phosphatase activity were not perfectly correlated, suggesting some posttranscriptional regulation of the enzyme. Thus, expression of a partly activated PKG2 renders osteoblasts largely resistant to dexamethasone-induced suppression of proliferation, differentiation, and survival, and it restores Wnt/β-catenin pathway–related genes in glucocorticoid-exposed cells.

### Cinaciguat improves bone formation, cortical thickness, and bone strength in male WT mice treated with dexamethasone.

Since cinaciguat decreased dexamethasone toxicity in primary osteoblasts, we tested the drug in a GIOP model in adult male C57BL/6Hsd mice. We injected 13-week-old mice with 2.5 mg/kg dexamethasone s.c. every other day for 5 weeks. This dexamethasone dose did not affect fasting serum glucose concentrations, glucose tolerance, body weight, or femur length of the mice (Supplemental Figures 2 and 3). Cinaciguat was used at a dose of 10 μg/kg daily and also did not alter weight or femur length (Supplemental Figure 3).

**Figure 3:**
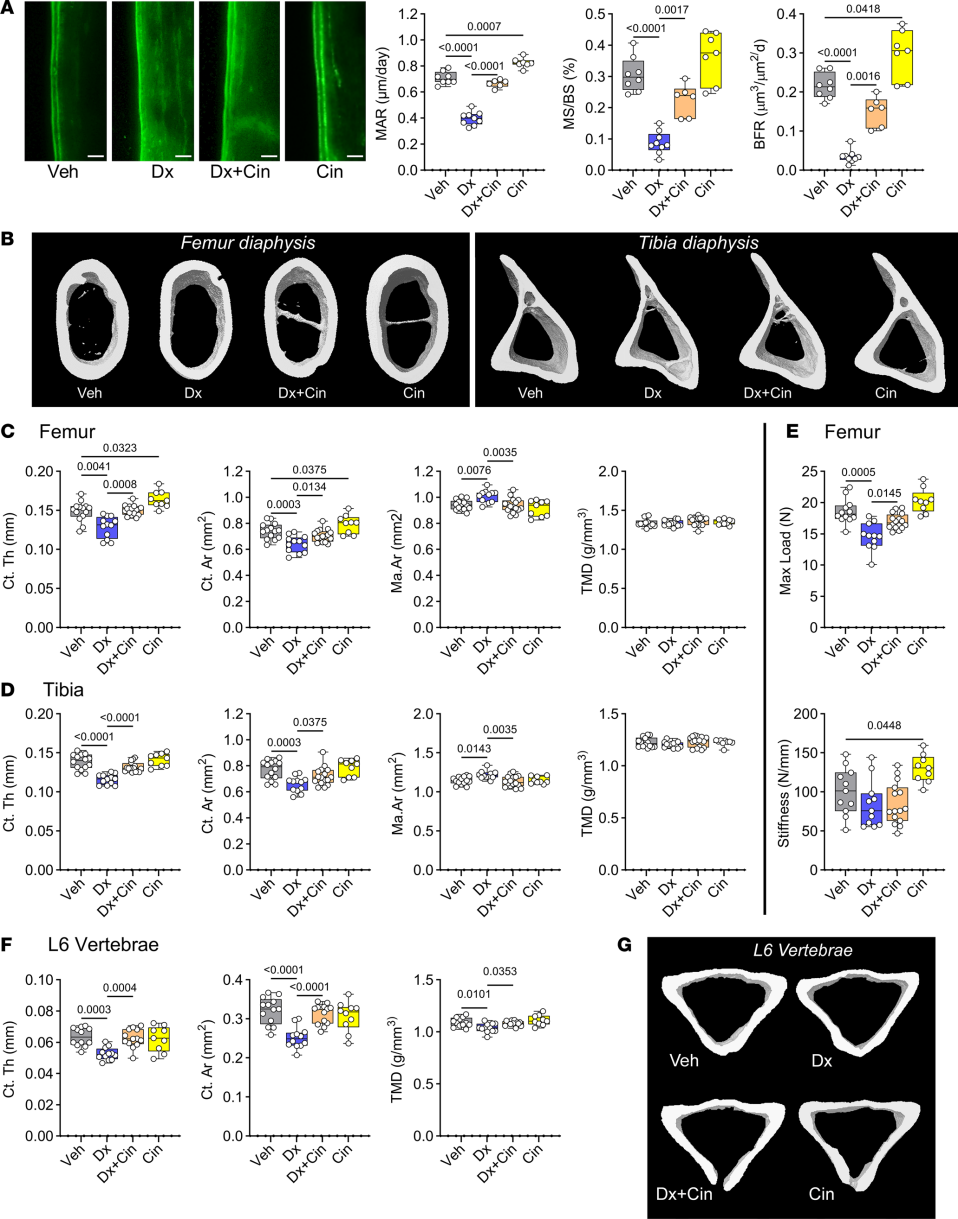
Cinaciguat improves bone formation, cortical thickness, and bone strength in male WT mice treated with dexamethasone. Thirteen-week-old male C57BL/6Hsd mice were treated with vehicle (Veh) or 2.5 mg/kg dexamethasone (Dx) by s.c. injection on alternate days for 5 weeks; some mice received cinaciguat 10 μg/kg daily s.c. in addition to dexamethasone (Dx+Cin), or cinaciguat alone (Cin). To label newly formed bone, all mice were injected with calcein 8 days and 2 days prior to euthanasia. (**A**) Double calcein labeling of endocortical bone in the femur middiaphysis was analyzed on longitudinal sections (*n* = 7–9 mice per group) to determine mineral apposition rate (MAR), mineralizing surface (MS/BS), and bone-formation rate (BFR). Scale bar: 25 μm. (**B**) μCT images of cortical bone in the middiaphysis of the femur (left) and tibia (right). (**C** and **D**) Cortical thickness (Ct.Th), cortical bone area (Ct.Ar), medullary area (Ma.Ar), and tissue mineral density (TMD) were analyzed by μCT in the middiaphysis of the femur (**C**) or tibia (**D**), as described in Methods (*n* = 9–16 mice per group). (**E**) Maximal load and stiffness of femurs was measured by 3-point bending as described in Methods (*n* = 9–16 mice per group). (**F** and **G**) μCT analysis and 3D images of cortical bone in L6 vertebrae (*n* = 9–13 mice per group). The box-and-whisker box plots show medians and 25th to 75th percentiles; the indicated comparisons were by Welch ANOVA with Dunnet’s T3 test, except for Ct.Ar, Ma.Ar, and TMD, which showed equal variances and were analyzed by regular 1-way ANOVA with Holm-Šidák’s multiple-comparison test.

Prior to euthanasia at 18 weeks of age, the mice were injected with calcein twice, 6 days apart, to label newly formed bone. Dexamathasone treatment severely reduced mineral apposition rates (MAR), mineralizing surfaces (mineralizing surface/bone surface [MS/BS]), and bone-formation rates (BFR) on endocortical surfaces of the femur; cotreatment with cinaciguat completely restored MAR, and partly restored MS/BS and BFR to values found in vehicle-treated mice (Figure 3A). By itself, cinaciguat increased BFR by 38% compared with vehicle, as previously shown in intact female mice (Figure 3A) (16). Microcomputed tomography (μCT) analysis of femurs, tibiae, and vertebrae showed that dexamethasone treatment reduced cortical bone volumes, evident from a decrease in cortical thickness (Ct.Th) and bone area (Ct.Ar) (Figure 3, B–D, F, and G). Cotreatment with cinaciguat effectively protected the mice against dexamethasone-induced cortical bone loss in all 3 bones; in the absence of dexamethasone, cinaciguat increased Ct.Th and Ct.Ar in the femur, but this effect was not apparent in tibiae or vertebrae (Figure 3, B–D, F, and G). The increase in medullary area (Ma.Ar) of femurs and tibiae observed in dexamethasone-treated mice was reversed by cinaciguat, although cinaciguat alone had no effect on Ma.Ar. Neither dexamethasone nor cinaciguat affected tissue mineral density (TMD) in the long bones, but in the vertebrae, dexamethasone decreased TMD, and cotreatment with cinaciguat reversed this effect (Figure 3, C, D, and F).

Since dexamethasone treatment may affect bone mechanical properties, we performed 3-point bending tests with femurs to assess bone strength and stiffness. Corresponding to its effect on Ct.Th and Ct.Ar, dexamethasone reduced strength, the maximum load achieved before fracture of the femur diaphysis, by 21%, and cinaciguat treatment partially prevented this, improving strength by 12% (Figure 3E). Femoral stiffness did not differ significantly between dexamethasone- and vehicle-treated mice (Figure 3E). In mice treated with cinaciguat alone, stiffness was increased, and there was a trend toward increased maximum load compared with vehicle-treated mice (Figure 3E).

Dexamethasone did not affect trabecular bone parameters in femurs or vertebrae, with the exception of femoral trabecular thickness (Tb.Th), which was slightly decreased (Supplemental Figure 4, A and B). Cinaciguat increased bone mineral density (BMD) and produced a trend toward increased BV/TV and Tb.Th in femurs (Supplemental Figure 4, A and B).

Thus, cinaciguat effectively prevented dexamethasone-induced cortical bone loss in long bones and vertebrae. However, dexamethasone caused little or no change in trabecular bone volumes, consistent with the lack of trabecular bone loss in prednisolone-treated C57BL/6NHsd mice reported by other investigators (18–20).

### Col1a1-Prkg2^R242Q^ transgenic mice are protected from dexamethasone-induced cortical bone loss.

Since PKG2^R242Q^-expressing osteoblasts were resistant to the detrimental effects of dexamethasone, we compared the effects of dexamethasone in adult male Col1a1*-Prkg2^R242Q^* transgenic mice and their WT littermates, using the same protocol as described above. Body weight and femur length did not differ between WT and *Prkg2^R242Q^* transgenic mice, and they were not affected by dexamethasone (Supplemental Figure 5, A and B).

**Figure 4:**
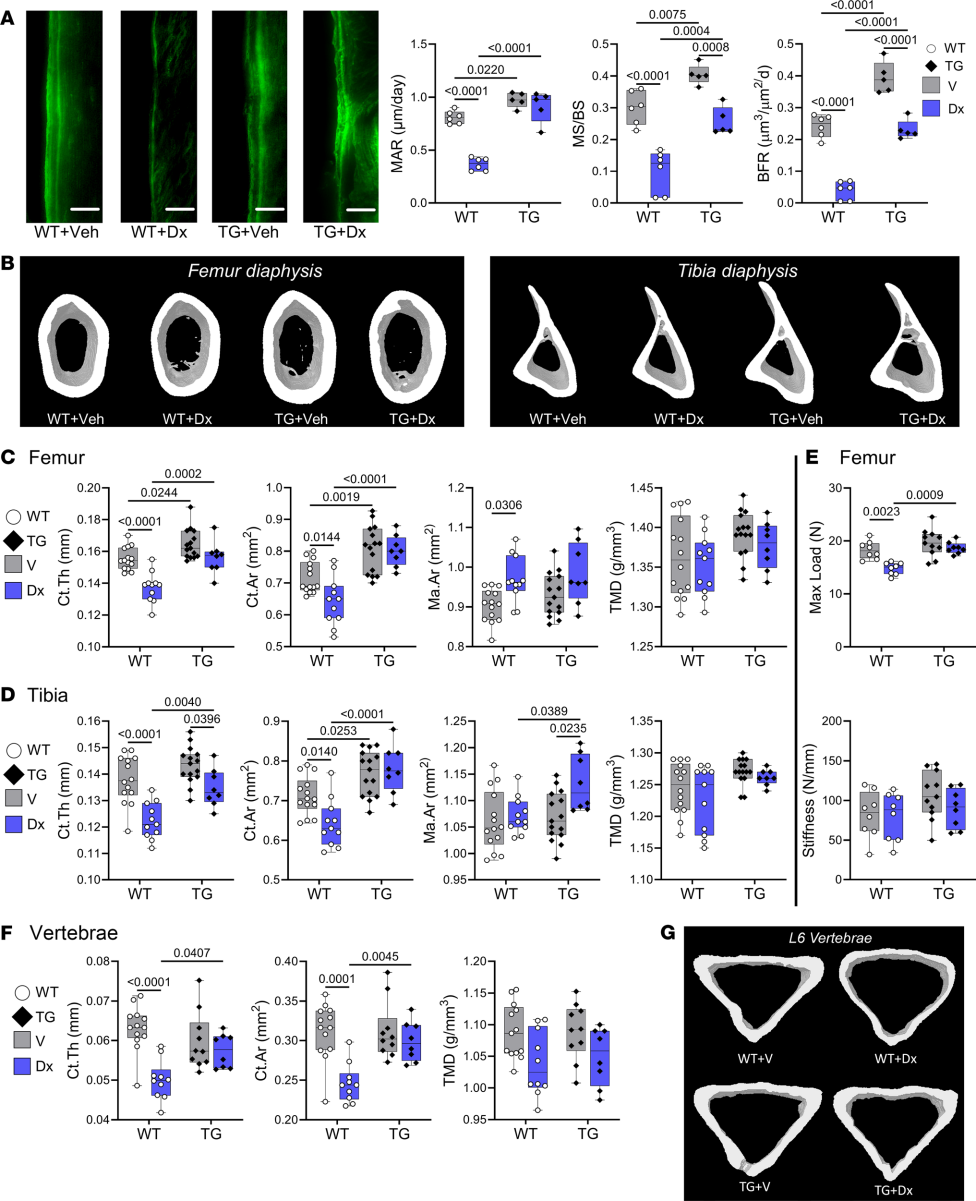
Col1a1-PKG2^R242Q^ transgenic mice are protected from dexamethasone-induced cortical bone loss. Osteoblast-specific Col1a1-PKG2^R242Q^ transgenic (TG) mice and WT littermates (WT) were treated with vehicle (V) or 2.5 mg/kg dexamethasone (Dx) by s.c. injection on alternate days for 5 weeks, starting at the age of 13 weeks. Eight and 2 days prior to euthanasia, mice were injected with calcein. (**A**) Mineral apposition rate (MAR), mineralizing surface (MS/BS), and bone-formation rate (BFR) were determined on endocortical surfaces of the femur middiaphysis as described in Figure 3A (*n* = 5–6 mice per group). Scale bars: 25 μm. (**B**) μCT images of cortical bone in the middiaphysis of the femur (left) and tibia (right). (**C** and **D**) Cortical thickness (Ct.Th), cortical bone area (Ct.Ar), medullary area (Ma.Ar), and tissue mineral density (TMD) were analyzed by μCT in the middiaphysis of the femur (**B**) or tibia (**C**), as described in Methods (*n* = 14–15 mice in the vehicle-treated groups and *n* = 8–11 in the dexamethasone-treated groups). (**E**) Maximum load and stiffness of femurs were measured by 3-point bending, as described in Methods (*n* = 8–11 mice per group). (**F** and **G**) μCT analysis and 3D images of cortical bone in L6 vertebrae (*n* = 8–13 mice per group). The box-and-whisker box plots show medians and 25th to 75th percentiles; the indicated comparisons were by 2-way ANOVA with Holm-Šidák’s multiple-comparison test.

The Col1a1*-Prkg2^R242Q^* transgenic mice showed higher basal MAR, MS/BS, and BFR compared with WT littermates (Figure 4A). In WT mice, dexamethasone severely reduced all 3 parameters, whereas in transgenic mice, the drug had no effect on MAR and only modestly reduced MS/BS and BFR to the levels observed in untreated WT mice (Figure 4A).

The *Prkg2^R242Q^* transgenic mice had higher femoral and tibial cortical bone volumes compared with their WT littermates, as previously described (15), but there was no difference in vertebral cortical volumes between the genotypes (Figure 4, B–D, F, and G; the trend toward higher tibial Ct.Th did not reach statistical significance). However, *Prkg2^R242Q^* transgenic mice were almost completely protected from the dexamethasone-induced femoral, tibial, and vertebral cortical bone loss that occurred in WT littermates (Figure 4, B–D, F, and G; only tibial Ct.Th was still slightly reduced by dexamethasone in transgenic mice). The transgenic mice were only partly protected from a dexamethasone-induced increase in femoral Ma.Ar (Figure 4C). TMD was not affected by dexamethasone and genotype (except a trend toward lower vertebral TMD in dexamethasone-treated WT mice; Figure 4F).

Mechanical testing by 3-point bending showed similar maximal load for vehicle-treated WT and transgenic mice, but consistent with their preserved cortical volume, the transgenic mice did not show any decrease in femoral maximal load after dexamethasone treatment, in contrast to WT littermates (Figure 4E). Femoral stiffness was not affected by dexamethasone or genotype (Figure 4E).

Compared with WT littermates, the *Prkg2^R242Q^* transgenic mice had higher femoral trabecular bone volumes and BMD, as previously described (15); they also showed higher BMD with a trend toward higher Tb.Th in L6 vertebrae (Supplemental Figure 6, A and B). Again, dexamethasone did not affect trabecular bone volumes, as discussed below.

### Cinaciguat increases osteoblast number and function, improves osteocyte survival, and restrains osteoclasts in the bones of dexamethasone-treated WT mice.

To determine which bone cells were targeted by cinaciguat in dexamethasone-treated mice, we examined BM stromal cells ex vivo, and measured osteoblast/osteocyte and osteoclast functional parameters in the serum and bones of treated mice.

**Figure 5:**
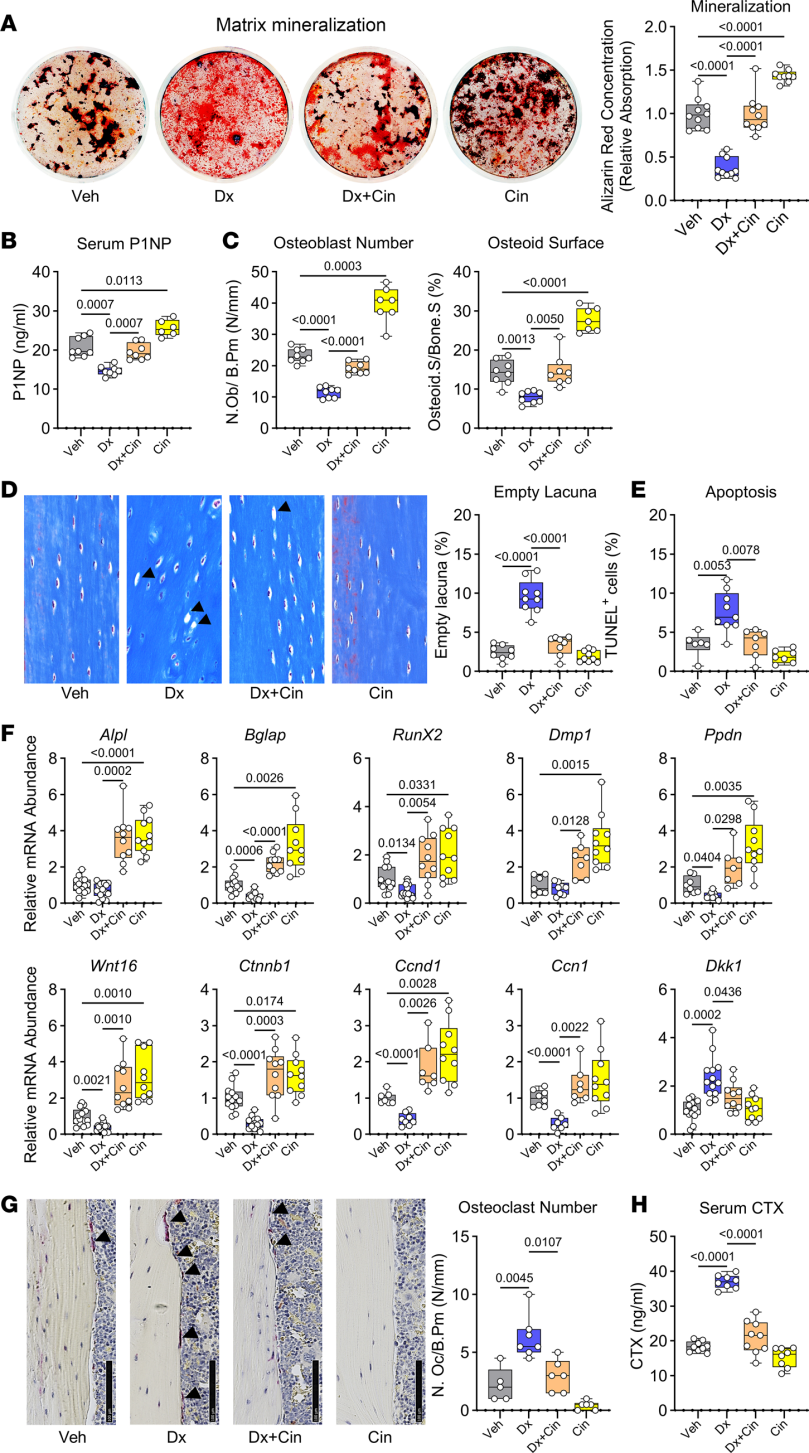
Cinaciguat increases osteoblast number and function, improves osteocyte survival, and restrains osteoclasts in the bones of dexamethasone-treated mice. Thirteen-week-old male C57BL/6Hsd mice were treated with vehicle (Veh) or dexamethasone (Dx) for 5 weeks, with some mice receiving cinaciguat in addition to dexamethasone (Dx+Cin) or receiving cinaciguat alone (Cin) as described in Figure 3. (**A**) Bone marrow stromal cells isolated from the treated animals were cultured in osteogenic differentiation medium containing ascorbate, β-glycerolphosphate, and 10 nM dexamethasone; mineralization was assessed by alizarin red staining after 21 days (without in vitro addition of cinaciguat or higher dexamethasone concentrations). Total calcium deposition was measured colorimetrically, with the mean of the vehicle-treated group assigned a value of one (*n* = 9–10 mice per group). (**B**) Procollagen-1 N-terminal peptide (P1NP) concentration in serum was measured by ELISA (*n* = 6–8 mice per group). (**C**) Osteoblast number and osteoid surface were determined on endocortical surfaces of the tibia on trichrome-stained longitudinal sections (*n* = 7–8 mice per group). (**D**) Empty lacunae (arrowheads) in the cortical bone (40× magnification) were counted on trichrome-stained tibial sections; results are expressed as percentage of empty/total lacunae (*n* = 8–9 mice per group). (**E**) Apoptotic osteocytes in cortical bone were detected by TUNEL staining on tibial sections (*n* = 6–9 mice per group). (**F**) Relative mRNA abundance of the indicated osteoblast- and Wnt-related genes was quantified in tibial cortical bone by RT-qPCR and normalized to 18S rRNA, with the mean ΔCt value obtained in untreated animals assigned a value of 1 (*n* = 9–14 mice per group, except *Dmp1, Ppdn, Ccnd1* and *Ccn1* with *n* = 7–10 mice per group; gene names as in Figure 1, E and F). (**G**) TRAP^+^ osteoclasts (arrowheads) were counted on tibial endocortical surfaces (photographs taken with 40× magnification; scale bar: 100 µm) (*n* = 5–7 mice per group). (**H**) C-terminal telopeptide (CTX) concentration in serum was measured by ELISA (*n* = 8–9 mice per group). The box-and-whisker box plots show medians and 25th to 75th percentiles; the indicated comparisons were by Welch ANOVA with Dunnett’s T3 test.

BM stromal cells isolated from dexamethasone-treated animals and cultured in osteoblast differentiation medium showed reduced mineralization capacity; calcium deposition in the extracellular matrix was measured by alizarin red staining, and the differentiation medium contained 10 nM dexamethasone in all groups (Figure 5A), since low glucocorticoid concentrations are required for optimal osteoblastic differentiation (21). BM stromal cells from mice cotreated with dexamethasone and cinaciguat had similar mineralization capacity as cells from vehicle-treated mice, whereas cells from mice treated with cinaciguat alone showed increased mineralization (Figure 5A). In these experiments, cinaciguat was present only in vivo and was not added to the culture medium in vitro. Thus, cinaciguat treatment preserved the osteoblastic differentiation potential of BM stromal cells in dexamethasone-treated mice.

The serum concentration of procollagen type I N-terminal propeptide (P1NP) was significantly suppressed in dexamethasone-treated mice, indicating suppression of osteoblast activity, but cinaciguat cotreatment increased serum P1NP concentrations almost to control values, and cinaciguat alone increased P1NP compared with vehicle-treated mice (Figure 5B). Consistent with these results, osteoblast numbers and osteoid surface were depressed in the dexamethasone-treated mice, but cinaciguat cotreatment restored both parameters to control levels, and cinaciguat alone increased osteoblast numbers and osteoid surfaces (Figure 5C).

Osteocytes are the most abundant (>90%) and longest-living cells in bone, responsible for mineral deposition and maintenance of bone structure; glucocorticoids can induce osteocyte apoptosis, and the latter correlates with deterioration of bone strength (22, 23). The cortical bone of dexamethasone-treated mice showed an increased number of empty lacunae, indicating loss of osteocytes, and increased numbers of apoptotic osteocytes detected by TUNEL staining; cinaciguat cotreatment reduced the number of empty lacunae and apoptotic osteocytes back to control values (Figure 5, D and E, and Supplemental Figure 7A).

Dexamethasone treatment significantly decreased osteoblast differentiation–related (*Bglap, Runx2, Ppdn*) and Wnt/β-catenin–related (*Wnt16, Ctnnb1, Ccnd1, Ccn1*) mRNA expression, while increasing expression of the Wnt inhibitor *Dkk1* in tibial diaphyses (reflecting largely osteocytes in cortical bone) (Figure 5F). In the absence or presence of dexamethasone, cinaciguat treatment increased osteoblast differentiation– and Wnt/β-catenin–related gene expression to levels higher than those found in control mice, and in the presence of dexamethasone, cinaciguat returned *Dkk1* mRNA back to control levels (Figure 5F). These data suggest that cinaciguat increased Wnt/β-catenin signaling in osteoblasts and osteocytes, reversing the negative effects of dexamethasone.

The number of osteoclasts on endocortical surfaces and the serum concentration of C-terminal collagen cross-links (CTX, indicating collagen degradation) increased in dexamethasone-treated mice, but cinaciguat reduced both parameters back to control values; there were trends toward reduced osteoclast number and CTX concentration in mice treated with cinaciguat alone, but they did not reach significance (Figure 5, G and H). Thus, cinaciguat restrained osteoclast number and collagen degradation in dexamethasone-treated mice.

### Col1a1-PKG2^R242Q^ transgenic mice are protected from dexamethasone-induced osteoblast loss and show improved osteocyte survival.

To determine whether increased PKG2 activity in cells of the osteoblast lineage could mimic the effects of cinaciguat in GIOP, we compared dexamethasone effects in Col1a1*-Prkg2^R242Q^* transgenic mice and their WT littermates.

**Figure 6:**
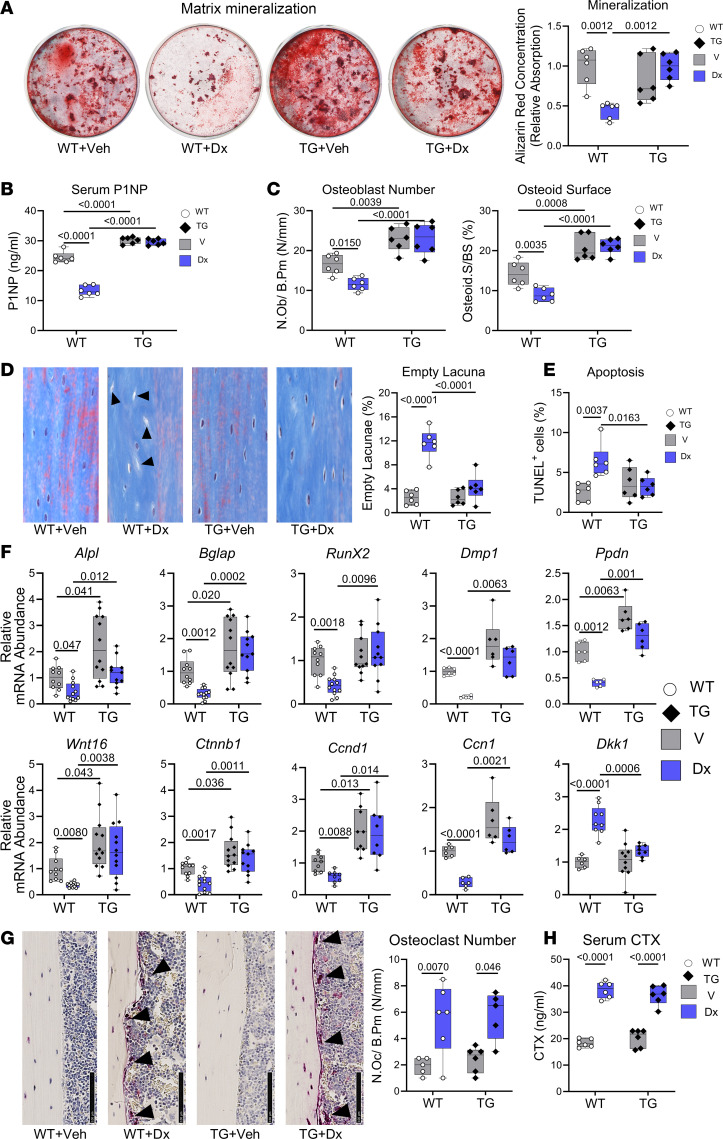
Col1a1-PKG2^R242Q^ transgenic mice are protected from dexamethasone-induced osteoblast loss and show improved osteocyte survival. Osteoblast-specific Col1a1-PKG2^R242Q^ transgenic (TG) mice and WT littermates (WT) were treated with vehicle (Veh) or 2.5 mg/kg dexamethasone (Dx) for 5 weeks, as described in Figure 4. (**A**) Bone marrow stromal cells isolated from the treated animals were cultured in osteogenic differentiation medium for 21 days, and mineralization was assessed by alizarin red staining as in Figure 5A (*n* = 6 mice per group). (**B**) P1NP serum concentration was measured by ELISA (*n* = 6 mice per group). (**C**) Osteoblast number and osteoid surface were determined on endocortical tibial surfaces as in Figure 5C (*n* = 6 mice per group). (**D**) Empty lacunae (arrowheads) in the tibial cortical bone (40× magnification) were counted as in Figure 5D (*n* = 6 mice per group). (**E**) Apoptotic osteocytes were detected by TUNEL staining as in Figure 5E (*n* = 6 mice per group). (**F**) Relative mRNA abundance of the indicated osteoblast- and Wnt-related genes was quantified in tibial cortical bone as described in Figure 5F (*n* = 11–12 mice per group, except for *Dmp1, Ppdn, Ccn1,* and *Dkk1* with *n* = 6–9 per group; gene names as in Figure 1, E and F). (**G**) TRAP-^+^ osteoclasts (arrowheads) were counted on tibial endocortical surfaces (photographs taken with 40× magnification scale bar: 100 µm) as in Figure 5G (n = 5-6 mice per group). (**H**) CTX serum concentration was measured by ELISA (*n* = 6–7 mice per group). The box-and-whisker box plots show medians and 25th to 75th percentiles; the indicated comparisons were by 2-way ANOVA with Holm-Šidák’s multiple-comparison test (**B**–**E**, and **H**) or by Welch ANOVA with Dunnett’s T3 test (**A**, **F**, and **G**).

Mineralization capacity of BM stromal cells isolated from Col1a1*-Prkg2^R242Q^* transgenic mice was similar to that of cells from WT mice, but it was not affected by dexamethasone treatment, in contrast to the decreased calcium deposition by cells from dexamethasone-treated WT mice (Figure 6A). Col1a1*-Prkg2^R242Q^* transgenic mice had higher serum P1NP concentrations, osteoblast numbers, and osteoid surfaces than WT mice; dexamethasone had no effect in transgenic mice, while decreasing all 3 parameters in WT littermates (Figure 6, B and C). The percentages of empty lacunae and TUNEL^+^ apoptotic osteocytes were similar in cortical bone sections of WT and transgenic mice, but dexamethasone increased empty lacunae and apoptotic cells only in WT mice (Figure 6, D and E, and Supplemental Figure 7B). The transgenic mice also showed higher *Alpl,* *Bglap, Ppdn, Wnt16, Ctnnb1,* and *Ccnd1* mRNA expression (and a trend toward higher *Dmp1* and *Ccn1* expression) in tibial diaphyses compared with WT littermates, and they were completely protected from the dexamethasone-induced suppression of these osteoblast differentiation– and Wnt/β-catenin–related genes (Figure 6F). Col1a1*-Prkg2^R242Q^* transgenic mice expressed similar amounts of *Dkk1* mRNA as WT mice, but again, they were protected from the dexamethasone-induced increase in the Wnt inhibitor (Figure 6F), suggesting that constitutive activation of PKG2 preserved Wnt signaling in the presence of dexamethasone. However, the effect of dexamethasone on endocortical osteoclast numbers and serum CTX concentrations did not differ between WT and transgenic mice, indicating that PKG2 activation in osteoblasts was not sufficient to restrain the dexamethasone-induced increase in osteoclasts (Figure 6, G and H).

### Cinaciguat prevents the inhibitory effects of dexamethasone on β-catenin expression and promotes nuclear β-catenin accumulation.

Glucocorticoids inhibit the Wnt/β-catenin pathway at multiple levels, including reduction of β-catenin mRNA and protein and inhibition of β-catenin–dependent transcription of target genes (2, 24–26). Glucocorticoids induce β-catenin degradation via inhibition of AKT and activation of glycogen synthase kinase-3β (GSK-3β), which targets β-catenin for the proteasome; Akt normally suppresses GSK-3β by phosphorylating an inhibitory site (27).

**Figure 7:**
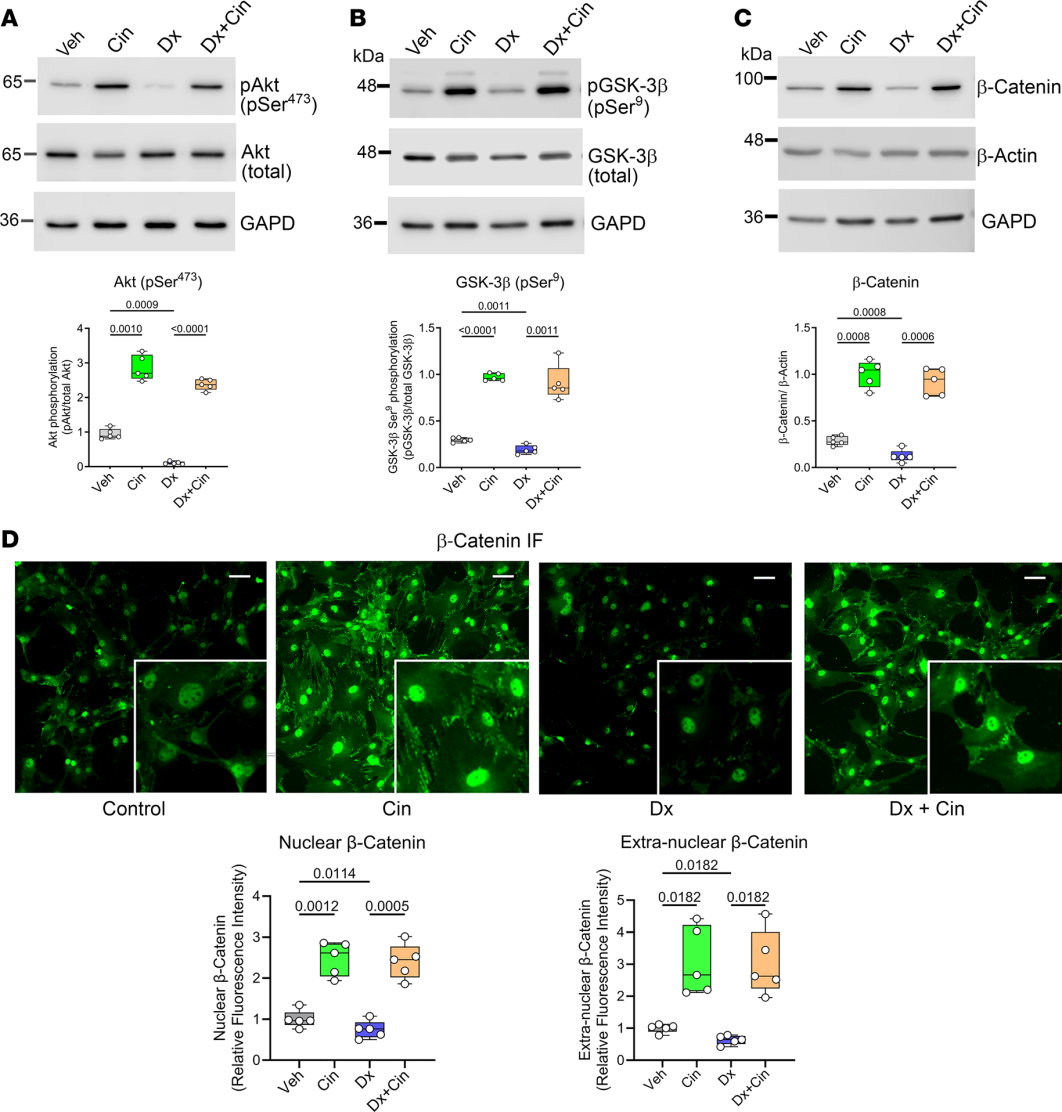
Cinaciguat prevents the inhibitory effects of dexamethasone on β-catenin expression and promotes nuclear β-catenin accumulation. Primary osteoblasts isolated from WT C57BL/6Hsd mice were treated with vehicle (Veh), cinaciguat (Cin, 1μM), dexamethasone (Dx, 1 μM), or Dx+Cin in medium containing 1% FBS. Treatment was for 1 hour (**A** and **B**), 24 hours (**C**), or 6 hours (**D**). (**A**) Akt activation was assessed by Western blotting using an antibody specific for Akt phosphorylated on Ser^473^, with total Akt and GAPD serving as loading controls. (**B**) GSK-3β inhibition was assessed by blotting with an antibody specific for GSK-3β phosphorylated on Ser^9^, with total GSK-3β and GAPD serving as loading controls. (**C**) Total β-catenin was assessed by Western blotting in whole cell lysates, with β-actin serving as a loading control. (**D**) Immunofluorescence staining of total β-catenin (green); photographs were taken at 20× (scale bar: 50 μm), and the insets are 40×. Nuclear β-catenin was quantified using CellProfiler. The box-and-whisker box plots show medians and 25th to 75th percentiles of 5 independent experiments; the indicated comparisons were by repeated-measures 1-way ANOVA with Geisser-Greenhouse correction (not assuming equal variances) and Holm-Šidák’s multiple-comparison test.

We found that dexamethasone treatment of primary osteoblasts reduced Akt phosphorylation on an activating site (Ser^473^) and reduced GSK-3β phosphorylation on the inhibitory Akt phosphorylation site (Ser^9^) (Figure 7, A and B). Correspondingly, total cellular content of β-catenin and nuclear β-catenin were decreased after dexamethasone exposure (Figure 7, C and D). In the presence or absence of dexamethasone, cinaciguat increased Akt pSer^473^ and GSK-3β pSer^9^, resulting in β-catenin accumulation (Figure 7, A–C). Cinaciguat also increased the amount of nuclear β-catenin > 2.5-fold in the presence or absence of dexamethasone (Figure 7D; extranuclear β-catenin was increased to a similar extent). Thus, cinaciguat prevented the dexamethasone-induced decline in total and nuclear β-catenin protein, consistent with its stimulatory effect on the β-catenin target genes *Ccnd1* and *Ccn1* (Figure 1F and Figure 5F).

## Discussion

Osteoporosis is a major side effect of glucocorticoid therapy and can be devastating, causing morbidity and even increased mortality due to spontaneous or low-impact fractures. GIOP is underrecognized and undertreated; first-line antiresorptive therapies are only partly effective and do not address the severe reduction in bone formation found in GIOP, but current bone-anabolic therapies have major limitations (4, 5, 7).

We identified the cGMP/PKG2 signaling pathway as a therapeutic target to prevent GIOP: the guanylyl cyclase-1 activator cinaciguat counteracted most of dexamethasone’s detrimenttal effects on bone in male mice; it recovered osteoblast number and bone formation, increased cortical bone volume and bone strength, and reduced osteoblast/osteocyte apoptosis. Osteoblast-specific expression of a constitutively active PKG2^R242Q^ similarly protected mice from bone-damaging effects of dexamethasone, but in contrast to systemic activation of cGMP/PKG signaling by cinaciguat, osteoblast-specific PKG2^R242Q^ expression did not reverse the glucocorticoid-induced increase in osteoclasts. Cinaciguat likely reduced osteoclast number and activity by activating PKG1, since the latter directly inhibits osteoclast differentiation and resorptive activity (28–30). However, our findings suggest that the bone-protective effects of genetic or pharmacological PKG2 activation in GIOP are primarily though preservation of osteoblast and osteocyte functions, rather than through inhibition of osteoclasts.

Osteoclast versus osteoblast contributions to GIOP are difficult to quantify, but an increase in bone resorption may dominate early on after initiating glucocorticoids (within 1 week in mice), whereas a decrease in bone formation supervenes during long-term administration (>4 weeks in mice) (2, 4, 31, 32). The resistance of osteocyte-specific RANKL-KO mice to glucocorticoid-induced cortical bone loss suggests an important role of RANKL-stimulated osteoclasts, but the unexplained low cortical bone volume of untreated RANKL-KO mice makes it difficult to quantitate osteoclast contributions (18).

Male Col1a1*-Prkg2^R242Q^* transgenic mice showed increased osteoblast numbers and bone formation parameters associated with increased trabecular and cortical bone volumes in femurs but with similar osteoclast parameters compared with WT mice, as previously reported (15). Correspondingly, pharmacological PKG2 activation by cinaciguat stimulated osteoblastic bone formation and increased cortical bone volumes, with a trend toward increased trabecular volumes in femurs of WT mice. PKG2 activation increases osteoblast proliferation via Src and ERK activation and positively regulates osteoblastic differentiation via stimulation of nuclear β-catenin signaling (15, 16, 28–30). We previously demonstrated bone-anabolic effects of cinaciguat in 2 mouse models of osteoporosis with predominantly trabecular bone loss: in female ovariectomized mice and in male mice with type 1 diabetes, cinaciguat prevented trabecular bone loss by improving bone formation and reducing excess osteoclast activity (14, 16). Here we show bone-protective effects of cinaciguat in a mouse model of GIOP, where suppression of bone formation and cortical bone loss predominated. Interestingly, pharmacological or genetic PKG2 activation also prevented dexamethasone-induced cortical bone loss in L6 vertebrae, although vertebral trabecular bone volumes were not affected in the absence or presence of dexamethasone.

One of the major mechanisms whereby glucocorticoids inhibit osteoblastogenesis and osteoblast/cyte functions is by interfering with Wnt/β-catenin signaling; glucocorticoids increase expression of Wnt antagonists, decrease Wnt ligands, and reduce nuclear β-catenin (8, 10–12, 24, 26, 27). Consistent with a central role of Wnt signaling in skeletal homeostasis and GIOP, mice with osteoblast or osteocyte-specific *Dkk1* KO and mice with *Wnt10b* transgenic overexpression are completely protected from GIOP, with preservation of osteoblast and bone formation parameters; however, these studies measured glucocorticoid effects only in trabecular bone (10, 11). Mice with osteoblast-specific overexpression of Wnt16 are partly protected from prednisolone-induced trabecular but not cortical bone loss, with partial preservation of BFR and no changes in osteoclasts (9). In contrast, mice with global *Sost* KO are fully protected from prednisolone-induced cortical and trabecular bone loss due to a reduction in osteoclast number and activity despite reduced bone formation; thus, sclerostin is required for glucocorticoid actions on bone resorption and not formation (12). However, the markedly altered bone microarchitecture of *Sost* KO and *Dkk1* KO mice at baseline may alter the bone microenvironment and bias the response to glucocorticoids. Moreover, strain-, age-, sex-, and dose-dependent differences of glucocorticoid actions in trabecular and cortical bone make it difficult to compare GIOP models across mice with different genetic modifications (20).

We found that dexamethasone increased *Dkk1* and reduced *Wnt16* mRNA in primary osteoblasts and bones of WT mice, with pharmacological or genetic PKG2 activation restoring expression of both genes to control levels. We propose that *Dkk1* and *Wnt16* regulation by cGMP/PKG2 contribute to the protective effects of cGMP/PKG2 signaling in GIOP (9, 11).

In canonical Wnt signaling, Wnt ligands bind to their receptors LRP5/6 and Frizzled, leading to inhibition of β-catenin ubiquitination and degradation, so that newly synthesized β-catenin can translocate to the nucleus and induce target genes, including *Ccnd1* (cyclinD1) and *Ccn1* (27, 33). We found that dexamethasone treatment downregulated *Cnnb1* (β-catenin) as well as *Ccnd1* and *Ccn1* mRNA in primary osteoblasts and bones of WT mice, but these effects were prevented by cinaciguat treatment or *Prkg2^R242Q^* transgene expression. Dexamethasone also decreased total and nuclear β-catenin protein in osteoblasts, consistent with the results of other workers (24–26). Cinaciguat reversed dexamethasone’s effects on total and nuclear β-catenin in part by inactivating GSK-3β, which normally phosphorylates and destabilizes β-catenin. We have shown previously that PKG2 activation in osteoblasts can directly and indirectly (via Akt) inactivate GSK-3β via phosphorylation on Ser^9^ (34). Direct pharmacological inhibition of GSK-3β is known to attenuate glucocorticoid-induced bone loss in rats (35). Although Wnt signaling in bone is not exclusively mediated by GSK-3β regulation of β-catenin, global GSK3β haploinsufficiency in mice leads to an increase in β-catenin, bone formation, and bone volumes, whereas postnatal deletion of β-catenin in osteoblast-lineage cells leads to low bone mass with reduced bone formation and increased resorption (36–38). Together, these data suggest that cinaciguat’s beneficial effects in GIOP are at least partly explained by GSK-3β inhibition and restoration of β-catenin expression in glucocorticoid-treated mice and osteoblasts.

Osteocyte apoptosis is a central feature of GIOP in humans and mice (2, 39, 40). Viable osteocytes inhibit osteoclast activity via secreted factors and support bone formation via release of anabolic signals such as NO, PGE2, ATP, and Wnt ligands, which directly stimulate osteoblast proliferation, differentiation, and matrix synthesis (41). Apoptotic osteocytes release agents, including RANKL, that induce osteoclasts to remodel damaged bone (41). The mechanisms whereby dying osteocytes suppress bone formation are not fully established but include lack of the anabolic signals, as well as release of Wnt inhibitors, as occurs in GIOP and during mechanical unloading (11, 23, 41, 42). These effects of osteocytes have been elegantly evaluated by Tatsumi et al. (23). After induction of osteocyte death by injection of diphtheria toxin into mice with osteocyte-specific expression of the diphtheria toxin receptor, they found that the RANKL/OPG ratio in bone increases, osteoclasts are recruited to empty lacunae, and bone formation and *Bglap* mRNA decrease (23). The decreased bone formation occurs within 1 week and without loss of osteoblasts, indicating communication between dying osteocytes and osteoblasts. These events lead to cortical thinning and decreased bone strength; however, Ct.Th and bone quality are restored 3 months later, as new osteocytes differentiate from osteoblasts (23). Our results indicate that PKG activation prevents glucocorticoid-induced osteocyte apoptosis and increases osteoblast proliferation and differentiation, potentially accelerating the replacement of apoptotic osteocytes and thereby preserving bone formation and strength in the presence of dexamethasone.

Previous workers have reported that the NO donor nitroglycerin ameliorates glucocorticoid-induced bone loss in rats; the mechanism was not examined, but the effect was attributed to osteoclast inhibition by NO (43). Our data suggest that NO acts through guanylyl cyclase stimulation and PKG activation. Epidemiological data suggest bone-protective effects of NO-generating nitrates with reduced fracture risk in older humans, and nitrates increase bone formation markers but without improving BMD in postmenopausal women (44–47). Nitrates are limited by the development of tolerance and bioconversion-dependent oxidative stress, however, and other cGMP-elevating agents may be better suited as bone-anabolic agents (13, 48).

Parathyroid hormone and antisclerostin antibodies improve BFRs, bone volumes, and bone strength in rodent models of GIOP (22, 49). Parathyroid hormone analogs improve BMD more effectively than antiresorptive agents in clinical trials of GIOP, with limited data suggesting reduced fracture risk (6). While parathyroid hormone analogs are FDA approved for the treatment of GIOP, their use is limited by cost, acceptance, and duration of efficacy (4). Current guidelines recommend against use of the antisclerostin antibody romosozumab in GIOP, due to potential harm from an increased risk of cardiovascular events (5). Thus, new treatment options for GIOP are needed.

We should note several limitations to our study. First, C57BL/6NHsd mice are relatively resistant to GIOP, and dexamethasone induced only cortical without trabecular bone loss, consistent with published reports in this strain (18–20). However, C57BL/6NHsd mice faithfully recapitulate glucocorticoid-induced cortical bone loss due to low bone turnover, with decreased bone strength, transient increase in osteoclasts, osteoblast dysfunction, and osteocyte apoptosis, as seen in humans (20). Longer-term treatment of older C57BL/6NHsd mice with glucocorticoids reduces trabecular bone volumes in vertebrae and long bones but is associated with generalized weight loss (50, 51). We examined only male mice, which are known to be more sensitive to GIOP than females (20, 52). Finally, we examined only 1 time point after dexamethasone administration, and we performed only limited gene expression studies. Advantages of our GIOP mouse model are that the mice were fully grown, did not show glucocorticoid-induced weight changes, and did not develop glucose intolerance, which could have confounded results. Moreover, we used clinically relevant doses of dexamethasone (2.5 mg/kg every other day in mice corresponds to ~0.1 mg/kg/d in humans). Clinical development of cinaciguat for treatment of severe heart failure was discontinued due to its blood pressure–lowering effect; however, at the dose we used to prevent GIOP in the mice, cinaciguat had no effects on systolic blood pressure (16). Riociguat and vericiguat are related guanylyl cyclase-1 stimulators, which are FDA approved for treatment of pulmonary hypertension and heart failure, respectively; other guanylyl cyclase stimulators are in clinical development (53).

In conclusion, PKG2 activation effectively prevented glucocorticoid-induced bone loss by protecting cells of the osteoblast lineage from loss of osteoprotective Wnt/ β-catenin signaling. Existing cGMP-elevating agents could be repurposed for the treatment of GIOP.

## Methods

### Sex as a biological variable.

We used male mice to avoid the possible influence of estrogens and because male C57BL/6NHsd mice have been shown to be more sensitive to GIOP than female mice (20, 52). However, we would expect our findings to be relevant for females as well.

### Chemicals and reagents.

BrdU (B5002), cinaciguat (SML1532), dexamethasone (D4902), calcein (C0875), p-nitrophenylphosphate (N2770), and alizarin red (A5533) were from MilliporeSigma. 3-(4, 5-Dimethylthiazol-2-yl)-2, 5-diphenyl tetrazolium bromide (MTT) was from Tocris Bioscience (catalog 5224). 8-(4-Chlorophenylthio)-cGMP (8-CPT-cGMP) was from BioLog. ELISAs for serum procollagen 1 N-terminal propeptide (PINP, MBS2500076) and CTX (MBS2607474) were from MyBioSource. All other chemicals were purchased from Sigma-Aldrich unless otherwise specified. Antibodies are summarized in Supplemental Table 1.

### Animal experiments.

Mice were housed 3–5 animals per cage in a temperature-controlled environment with a 12-hour light/dark cycle; they had ad libitum access to water and chow (Teklad Rodent Diet, 8604). Two cohorts of 12-week-old male C57BL/6NHsd mice were purchased from Envigo at different times. After 1 week of acclimatization, mice weighing 25–26 g were randomly divided into 4 groups and treated for 5 weeks as follows: (a) vehicle (3 % ethanol in normal saline, 0.1 mL s.c. on alternate days); (b) dexamethasone (Dx, 2.5 mg/kg s.c. on alternate days); (c) dexamethasone + cinaciguat (Dx+Cin; cinaciguat HCl, 10μg/kg s.c., daily); and (d) cinaciguat alone (Cin). The cinaciguat dose does not significantly reduce systolic blood pressure (<10 mmHg) and is sufficient to prevent bone loss in ovariectomized mice (16). In the first cohort, a total of 11 mice were assigned to groups (a) to (c); however, 2 mice from group (a) and 4 mice from group (b) were excluded prior to completing treatment because they were either injured by aggressive cage mates or they had to be housed singly due to their aggressive behavior. In the second cohort, 4–5 mice were assigned to groups (a) through (c), and 9 mice were assigned to group (d).

Transgenic mice with osteoblast-specific expression of a partly activated PKG2 (Col1a1-PKG2^R242Q^) were in a C57BL/6NHsd background and were previously characterized (15). At the time of weaning, male transgenic mice and their WT littermates were placed in cages together prior to genotyping; cages with 3–5 mice were randomly assigned to treatment with either vehicle or dexamethasone as described above, starting at 13 weeks of age.

In a subset of mice, an i.p. glucose tolerance test (IPGTT) was conducted 2 days before euthanasia. After a 5-hour fast, a glucose solution was injected i.p. (2 g/kg). Blood glucose concentrations were measured using about 10 μL of blood from a tail nick, just before the glucose injection (time 0) and at 15, 30, 60, 90, and 120 minutes following the injection. All mice were injected i.p. with calcein dissolved in 2% NaHCO_3_ (25 mg/kg) on day 8 and day 2 prior to euthanasia. Mice were euthanized at 18 weeks of age by CO_2_ intoxication and exsanguination between 8 a.m. and 11 a.m., approximately 24 hours after the last drug or vehicle injection.

### Cell culture and osteoblastic differentiation.

Primary osteoblasts and bone marrow stromal cells were isolated from long bones of male Col1a1-PKG2^R242Q^ transgenic and WT littermates mice at the age of 12–13 weeks as described (15, 54, 55). Primary osteoblasts were used passages 3–5; cells were grown in DMEM/F12 medium (Gibco/Life Technologies) supplemented with 10% FBS (Sigma-Aldrich) and penicillin (100 U/mL), streptomycin (100 μg/mL), and amphothericin (0.25 μg/mL) (Antibiotic-Antimycotic, Gibco/Life Technologies). Cells were treated with dexamethasone (2.5 μM, except where noted) and cinaciguat (1 μM) as indicated. For osteoblast differentiation, confluent cells were placed in medium supplemented with 0.3 mM ascorbate and 10 mM β-glycerolphosphate. Media and drugs (0.5 μM dexamethasone, 1 μM cinaciguat) were replaced on days 2, 4, and 6, and after 7 days; cells were fixed in 10% neutral buffered formalin and stained with p-nitrophenylphosphate to assess ALP activity; or RNA was extracted to determine osteoblast differentiation–associated gene expression as described below (14). ALP activity was normalized to protein concentration. Images were captured on an EPSON high-resolution scanner and analyzed with ImageJ (NIH).

Bone marrow–derived cells were subjected to RBC lysis and plated at 1 million cell/cm^2^ in 12-well plates in α-MEM supplemented with 10% FBS and antibiotic/antimycotic. Adherent bone marrow stromal cells were transferred 48 hours later to mineralization medium containing 0.3 mM ascorbate, 10 mM β-glycerolphosphate, and 10 nM dexamethasone. After 21 days, mineralized nodules were fixed in 10% neutral buffered formalin and stained with alizarin red as described (15). To determine total calcium deposition, alizarin red was extracted from washed cells using 10% acetic acid, which was neutralized with 10% ammonium hydroxide solution, and absorbance was measured at 405 nm.

### Cell proliferation, apoptosis, and MTT assay.

To assess proliferation by BrdU incorporation, cells were cultured on glass coverslips in medium containing 1% FBS and 200 μM BrdU for 24 hours. Cells were fixed in 4% formaldehyde and permeabilized with 1% Triton X-100, followed by treatment with DNase I and staining with anti-BrdU antibody (MilliporeSigma, B8434, 1:100 dilution) and a secondary TRITC-conjugated antibody (Invitrogen), as described (56).

To assess apoptosis, cleaved caspase-3 immunostaining was performed on primary osteoblasts plated on glass coverslips and treated for 18 hours with the indicated drugs in medium containing 1% FBS. Cells were fixed in 4% paraformaldehyde, permeabilized with 1% Triton X-100, and incubated with cleaved caspase-3–specific antibody (Cell Signaling Technology, CST 9661S, 1:100 dilution), followed by a secondary FITC-conjugated antibody (Invitrogen), as described (15). Nuclei were counterstained using Hoechst 33342, and the resulting images were analyzed using a Keyence BZ-X700 fluorescence microscope. The percentage of cells staining positive for BrdU or cleaved caspase-3 was determined by an observer blinded to the treatment of the cells, scoring at least 200 cells per condition.

For MTT reduction to formazan, osteoblasts were seeded in 96-well plates (2 × 10^3^ cells/well). Cells were treated with the indicated drugs in medium containing 1% FBS for 48 hours and received MTT (0.5 mg/mL) for the last 4 hours. Formazan crystals were dissolved in DMSO, and absorbance was recorded at 540 nm.

### Quantitative reverse transcription PCR (RT-qPCR).

The right tibia was harvested and quickly cleaned of surrounding soft tissue, including the periosteum; ~1 mm was cut off both ends; the bone marrow was flushed out with cold PBS; and the cortical bone shafts were snap frozen in liquid nitrogen. The frozen bones were pulverized in liquid nitrogen using a prechilled mortar and pestle, and total RNA was extracted using TRI reagent (Molecular Research Center). After reverse transcription (iScript cDNA Synthesis Kit, Biorad), PCR was performed in an Aligent MX3005P real-time PCR system using PowerUp SYBR Green Master Mix (Applied Biosystems, A25742) as described (14). Primer sequences are in Supplemental Table 2; all primers were tested with serial cDNA dilutions. Relative changes in mRNA expression were analyzed using the 2^−ΔΔCt^ method, with 18S and *Hprt* serving as internal references. Mean ΔCt values (gene of interest minus reference gene) measured in the vehicle-treated control group were assigned a value of 1.

### Western blots, immunofluorescence staining, and ELISA.

Western blots were generated using the antibodies described in Supplemental Table 1; horseradish peroxidase–conjugated secondary antibodies were detected by enhanced chemiluminescence (Pierce ECL substrate); densitometry scanning was performed with ImageJ (NIH).

For β-catenin immunofluorescence staining, cells were plated on glass cover slips and treated for 6 hours as indicated. Cells were fixed in 4% paraformaldehyde, permeabilized with 1% Triton X-100, and incubated with a β-catenin–specific antibody (CST 9582, 1:100 dilution), followed by a secondary FITC-conjugated antibody (Invitrogen). Nuclei were counter-stained with Hoechst 33342, and images were analyzed using a Keyence BZ-X700 fluorescence microscope and CellProfiler image analysis software (57).

Serum PINP and CTX concentrations were measured using commercial ELISA kits with standards according to the manufacturer’s protocol (MyBioSource).

### μCT.

Left femurs, tibiae, and vertebrae were fixed in 10% neutral buffered formalin for 24 hours and then stored in 70% ethanol at 4°C. The bones were scanned using a SkyScan 1076 (Bruker, Kontich, Belgium) μCT scanner at a voxel size of 9 μm, applying an electrical potential of 50 kV and current of 200 μA, and using a 0.5 mm aluminum filter (58). Scans were obtained over 360 degrees, with images captured every 0.8 degrees. Bones were kept hydrated by wrapping them in PBS-soaked tissue paper. A beam-hardening correction algorithm was applied before image reconstruction using Nrecon software (Bruker). DataViewer and CTAn software (Bruker) were used to orient bones longitudinally and to analyze bone morphometry, respectively. In the (distal) femoral and (proximal) tibial metaphysis, trabecular bone was identified by automatic contouring 0.44 to 1.32 mm proximal or distal to the growth plate, respectively; in L6 vertebrae, trabecular bone was identified by manual contouring from 0.99 to 1.71 mm above the caudal growth plate. An adaptive threshold (0.1788–1.668 g/cm^3^) was used to analyze trabecular bone. Cortical bone of the femoral or tibial diaphysis was identified by automatic contouring 4.4–5.2 mm proximal or distal to the growth plates, respectively, and analyzed using a fixed global threshold (0.542–1.668 g/cm^3^). The cortex of the L6 vertebra was identified by manual contouring from 0.63 to 0.81 mm proximal to the caudal growth plate and analyzed with the same threshold. Mineral density was determined by calibrating the CT-Analyzer histogram setting in reference to images derived from scanning 4 mm diameter hydroxyapatite phantom calibration rods of known mineral density (0.25 g/cm^3^ and 0.75 g/cm^3^). Three-dimensional images were generated from the volume of interest using CTAn and CTVox software (Bruker).

### Three-point bending.

To evaluate bone stiffness and strength, right femurs were tested to failure using a 3-point bending test on a Biomomentum V500cs (Biomomentum) instrument as described (59). Femurs were cleaned of soft tissue, wrapped in PBS-soaked tissue paper, and stored in –20°C. Before mechanical testing, bones were thawed and equilibrated to room temperature by submerging in PBS at room temperature for 2 hours. The femur was positioned horizontally on 2 supports separated by a distance of 6 mm, with the anterior surface upward, and the loading force was directed vertically to the midshaft of the bone at a displacement rate of 0.16 mm/s. Load and displacement were recorded with manufacturer-provided software. Strength was determined as the maximum load; stiffness was calculated as the slope of the linear portion of the load-displacement curve.

### Bone histomorphometry and analysis of stained bone sections.

After μCT analysis, the fixed femurs were subjected to KOH processing to destabilize collagen but retain the mineral, as described (60). Paraffin sections (8 μm) were prepared, and fluorescence images were captured for Calcein double labeling. Dynamic histomorphometry parameters were measured according to convention, as described previously (58, 61). In 2 dexamethasone-treated WT mice that showed only single calcein labeling, a minimum MAR value of 0.3 μm/d was used (61).

Fixed tibiae were decalcified in 10% EDTA solution at pH 7.4 for 5 days. Paraffin sections (5 μm) were subjected to Trichrome, TUNEL, and TRAP staining. A TUNEL staining kit was used per the manufacturer’s instructions (MilliporeSigma, S7100). Osteoclast numbers were estimated by TRAP staining of tibial sections as described (62). After paraffin removal, sections were treated with 0.1% Triton X-100 (5 minutes), rinsed, and incubated with 0.01% naphthol AS-MX phosphate and 0.05% Fast Red Violet LB Salt in 50 mM sodium tartrate and 90 mM sodium acetate (pH 5.0; 1 hour at 37°C). Images were captured with a Hamamatsu NanoZoomer 2.0 HT System and analyzed using NDP.view2 software. Three random areas of ~ 0.05 mm^2^ were selected per Trichrome- and TUNEL-stained cortical bone section to count the number of empty lacunae or TUNEL^+^ osteocytes, respectively; data were expressed as a percentage of total osteocytes. Osteoblasts were counted, and osteoid was measured on Trichrome-stained sections; osteoclasts were counted on TRAP-stained sections; endocortical surfaces were analyzed between 2 and 4.5 mm proximal to the growth plate on longitudinal sections. Histomorphometrical analyses were performed by an observer blinded to the genotype and treatment of the mice.

### Statistics.

GraphPad Prism 9 software was used for statistical analyses. All data sets were tested for normality using the Shapiro-Wilk test and for equality of variances by F test. Only data that passed both tests were analyzed by standard 1-way or 2-way ANOVA, and paired comparisons were made using the Holm-Šidák’s multiple-comparison test. Data that exhibited normality but not equality of variances were analyzed by a Welch 1-way ANOVA followed by Dunnett’s T3 test of multiple comparisons, and data from cell culture experiments were analyzed by repeated-measures 1-way or 2-way ANOVA with Geisser-Greenhouse correction. Data that did not exhibit normality were analyzed by the nonparametric Kuskal-Wallis test followed by a Dunn’s test of multiple comparisons. *P* < 0.05 was considered significant. Data are shown in box-and-whisker plots, with boxes extending from the 25th to the 75th percentiles and whiskers extending from minimum to maximum values; the line in the middle of the box is the median.

### Study approval.

All mouse procedures were approved by the UCSD IACUC (protocol no. S10121).

### Data and materials availability.

All raw data files for statistical analysis are available in the Supporting Data Values file. Mice generated for this manuscript are available with a UCSD Material Transfer Agreement.

## Author contributions

Study design was contributed by SPC and RBP. Study conduct was contributed by SPC, HK, SZ, and JAC. Data collection was contributed by SPC, HK, and SZ. Data analysis was contributed by SPC, RLS, and RBP. Data interpretation was contributed by SPC, HK, RLS, and RBP. Drafting manuscript was contributed by SPC and RBP. SPC and RBP take responsibility for the integrity of the data analysis.

## Supplementary Material

Supplemental data

Unedited blot and gel images

Supporting data values

## Version 1 06/17/2024

In-Press Preview

## Version 2 07/22/2024

Electronic publication
