# Supplementary material for: Protein kinase G2 activation restores Wnt signaling and bone mass in glucocorticoid-induced osteoporosis in mice
Source: JCI Insight. 2024 Jun 17;9(14):e175089. doi: 10.1172/jci.insight.175089 (PMC11383176; doi:10.1172/jci.insight.175089)
Supplement: Supplemental data [file jciinsight-9-175089-s086.pdf]

SUPPLEMENTAL MATERIALS

FOR:

Protein kinase G2 activation restores Wnt signaling and bone mass in glucocorticoid-induced osteoporosis in mice

Shyamsundar Pal China, Hema Kalyanaraman, Shunhui Zhuang, Justin A. Cabriales, Robert L. Sah, and Renate B. Pilz

Suppl. Figure 1

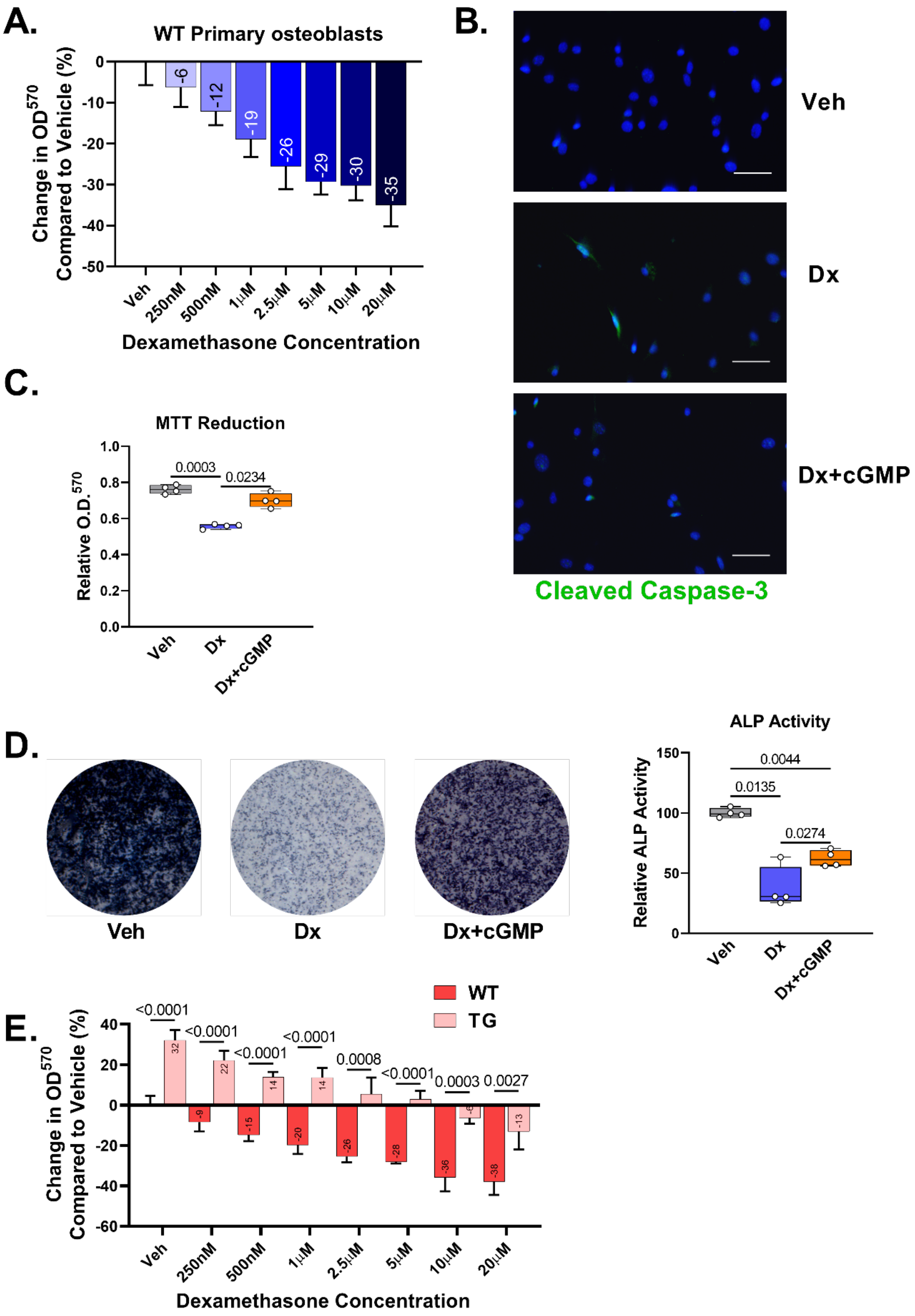

Supplemental Figure 1: Effect of increasing dexamethasone concentrations on MTT reduction in primary osteoblasts; 8-CPT-cGMP protection of osteoblasts from dexamethasone-induced reductions in survival, MTT reduction and alkaline phosphatase activity. A. Primary osteoblasts (POBs) from wild type C57BL/6NHsd mice cultured in medium containing 1% FBS were treated with vehicle (Veh) or the indicated dexamethasone concentration for 48 h; MTT was added for the last 4 h. MTT reduction to formazan was measured spectrophotometrically at 570 nm. **B.** Apoptosis was assessed by immunostaining for cleaved caspase-3 as described in Fig. 1B, after incubating POBs with 2.5 μM dexamethasone (Dx) for 18 h in the absence or presence of the cell-permeable cGMP analog 8-CPT-cGMP (cGMP, 100 μM) (bar = 25 μm). **C.** MTT assays were performed as in panel A, with 2.5 μM dexamethasone in the absence or presence of 100 μM 8-CPT-cGMP. **D.** Alkaline phosphatase (ALP) activity was measured as described in Fig. 1D, after 7 d culture in osteoblast differentiation medium in the absence or presence of 0.5 μM Dx and/or 100 μM 8-CPT-cGMP**. E.** POBs from wild type (WT, dark red) or Col1a1-PKG2^{R242Q} transgenic (TG, light red) mice were treated with increasing dexamethasone concentrations and MTT assays were performed as in panel A. Comparisons in C and D by repeated measures one-way ANOVA with Holm-Sidak's multiple comparisons test (n=4 independent experiments). Comparisons in E by two-way ANOVA with Sidak's multiple comparisons test (n=3 experiments).

Suppl. Fig. 2

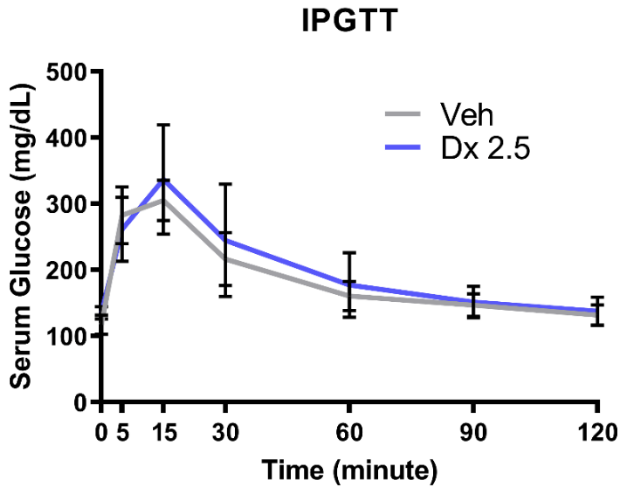

<u>Supplemental Figure 2:</u> Glucose tolerance test. Wild type mice were treated with vehicle or dexamethasone (2.5 mg/kg subcutaneously on alternate days) for five weeks, as described in Methods. To measure glucose tolerance, mice were injected intraperitoneally with 2g/kg of glucose and blood glucose concentrations were measured at the indicated times thereafter.

Suppl. Fig. 3

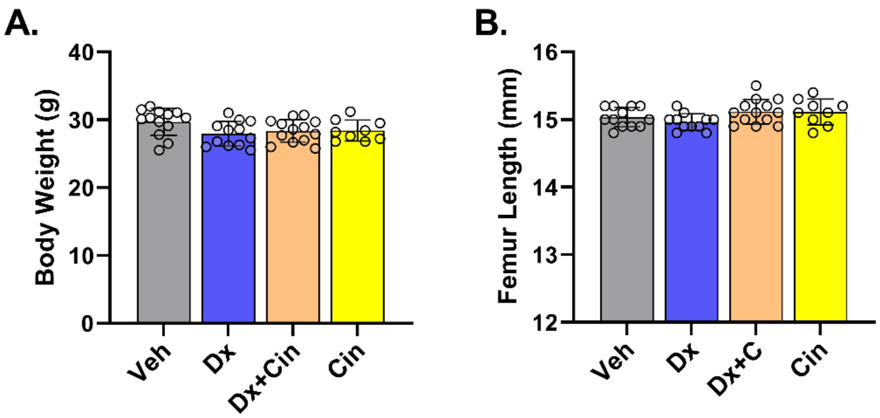

<u>**Supplemental Figure 3:**</u> **Body weights and femur lengths of dexamethasone-treated wild type mice**. Thirteen week-old male C57Bl/6Hsd mice were treated with vehicle or 2.5 mg/kg dexamethasone (Dx) by subcutaneous injection on alternate days for five weeks; some mice received cinaciguat 10 µg/kg daily subcutaneously in addition to dexamethasone (Dx+C) or cinaciguat alone (Cin). A. Mice were weighed prior to euthanasia. B. After euthanasia, femurs were dissected and disarticulated and femur lengths were measured by caliper.

Suppl. Fig. 4

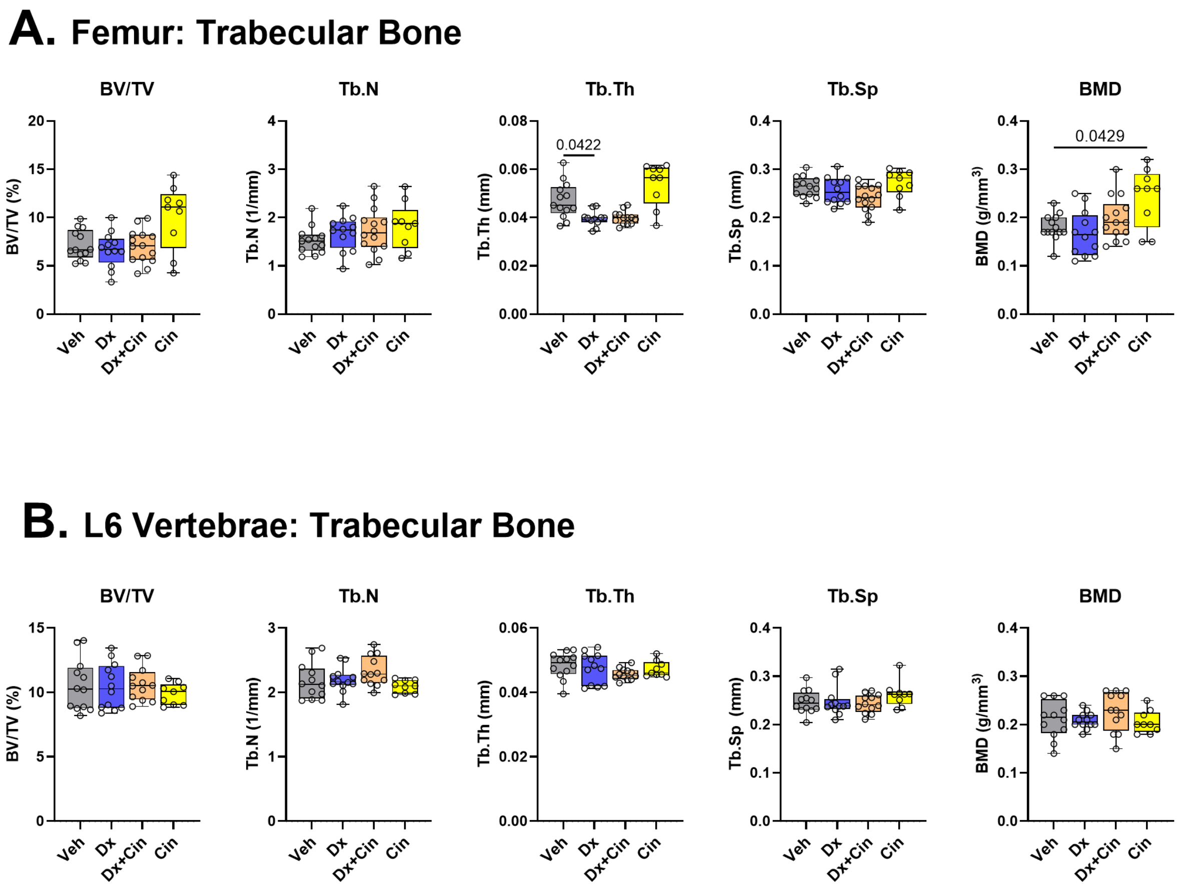

<u>Supplemental Figure 4:</u> Trabecular bone volumes in femurs and L6 vertebrae of wild type mice treated with dexamathasone and/or cinaciguat. Trabecular bone volumes were measured in femurs (A) and L6 vertebrae (B) of 18 week-old male C57Bl/6Hsd mice, treated with vehicle (Veh), dexamethasone (Dx), dexamethasone plus cinaciguat (Dx+Cin), or cinaciguat alone (Cin) for five weeks as described in Fig. 3. Bone volume fraction (BV/TV), trabecular number (Tb.N), trabecular thickness (Tb.Th), trabecular spacing (Tb.Sp) and bone mineral density (BMD) were analyzed by micro-CT in the metaphysis of the distal femur (A) or in L6 vertebrae (B), as described in Methods (n=9-14 mice per group). Comparisons by Welch-ANOVA with Dunnet's T3 test.

Suppl. Fig. 5

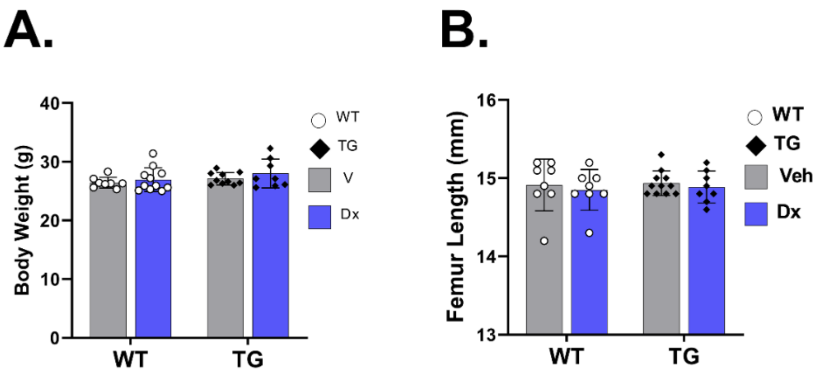

<u>Supplemental Figure 5:</u> Body weights and femur lengths of dexamethasone-treated wild type and transgenic mice. Osteoblast-specific Col1a1-PKG2^{RQ} transgenic mice (TG) mice and wild type litter mates (WT) were treated with vehicle or 2.5 mg/kg dexamethasone (Dx) by subcutaneous injection every alternate day for five weeks, starting at the age of thirteen weeks. A. Mice were weighed prior to euthanasia. B. After euthanasia, femurs were dissected and disarticulated and femur lengths were measured by caliper.

Suppl. Fig. 6

A. Femur: Trabecular Bone

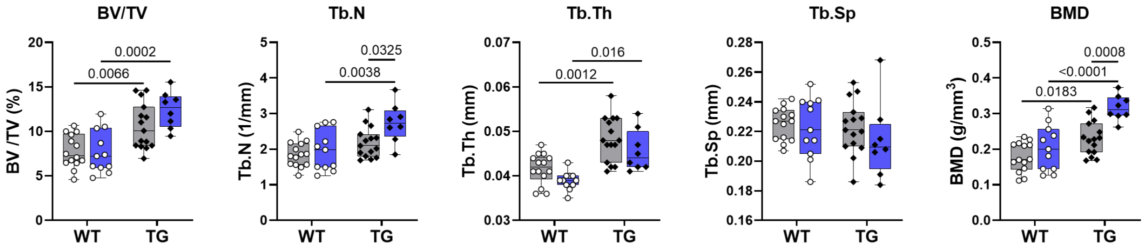

B. L6 Vertebrae: Trabecular Bone

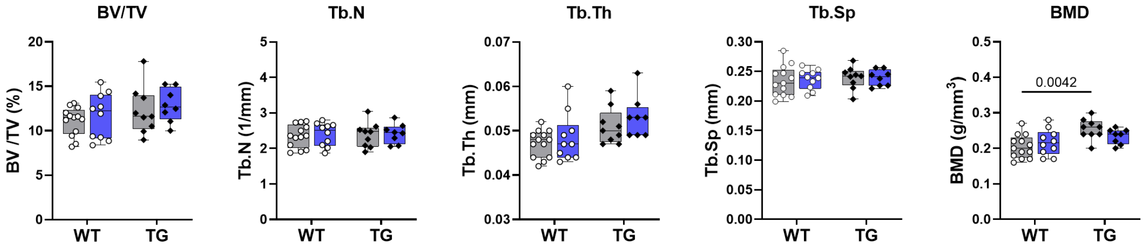

__**Supplemental Figure 6:**__ **Trabecular bone volumes in femurs and L6 vertebrae of wild type and Co1a1-PKG2^{R242Q} transgenic mice treated with vehicle or dexamathasone.** Trabecular bone volumes were measured in femurs (A) and L6 vertebrae (B) of 18 week-old male Co1a1-PKG2^{R242Q} transgenic mice and their wild type litter mates, treated with vehicle (Veh) or dexamethasone (Dx) for five weeks as described in Fig. 4. Bone volume fraction (BV/TV), trabecular number (Tb.N), trabecular thickness (Tb.Th), trabecular spacing (Tb.Sp) and bone mineral density (BMD) were analyzed by micro-CT in the metaphysis of the distal femur (A) or in L6 vertebrae (B), as described in Methods (n=8-15 mice per group). Comparisons by two-way ANOVA with Holm-Sidak's multiple comparisons test.

Suppl. Fig. 7

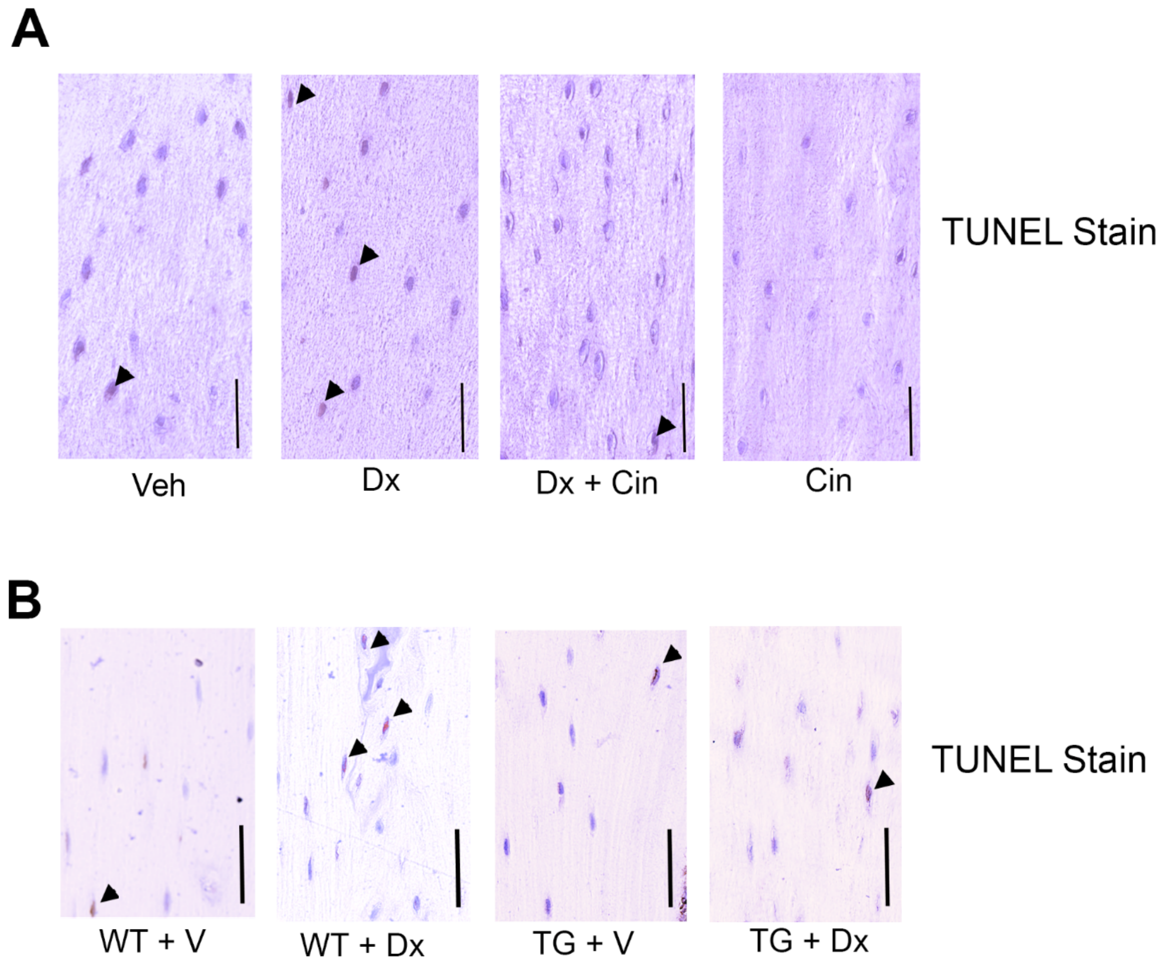

<u>Supplemental Figure 4:</u> TUNEL staining of cortical bone sections from wild type and Co1a1-PKG2^{R242Q} transgenic mice. (A) 18 week-old male C57Bl/6Hsd mice, treated with vehicle (Veh), dexamethasone (Dx), dexamethasone plus cinaciguat (Dx+Cin), or cinaciguat alone (Cin) for five weeks as described in Fig. 3. **(B)** 18 week-old male Co1a1-PKG2^{R242Q} transgenic mice and their wild type litter mates, treated with vehicle (Veh) or dexamethasone (Dx) for five weeks as described in Fig. 4. Tibial cortical sections were stained according to the TUNEL protocol, as described in Methods (bars, 50 μm). Arrow heads point to brown labeled nuclei, indicating apoptotic osteocytes.

Supplemental Table 1:
Antibodies used for Western blotting (WB) and immunofluorescence (IF)

Antibody Target	Catalog number	Source	Dilution for WB	Dilution For IF
β-Actin	SC-47778	Santa Cruz Biotech.	WB 1:5000	
Akt (pSer^{473})	9271	Cell Signaling	WB 1:1000	
Akt (total)	9272	Cell Signaling	WB 1:1000	
BrdU	B8434	Sigma-Aldrich		IF 1:100
β-Catenin	9582	Cell Signaling	WB 1:1000	
Cleaved caspase-3	9661	Cell Signaling		IF 1:100
GAPD	2118	Cell Signaling	WB 1:2000	
GSK-3β (pSer^{9})	9323	Cell Signaling	WB 1:1000	
GSK-3β (total)	610201	BD Biosciences	WB 1:1000	
FITC-anti-rabbit	111-095-003	Jackson Immunoresearch		IF 1:100
TRITC-anti-mouse	115-025-003	Jackson Immunoresearch		IF 1:100

<u>Suplemental Table 2:</u> Primers Used for Quantitative RT-PCR

Gene Abbrev.	Gene Name	Sense Primer (5'-3')	Anti-sense Primer (5'-3')
18S	18s Ribosomal RNA	GATCCATTGGAGGGCAAGTCT	CCAAGATCCAACTACGAGCTTT TT
Alpl	Alkaline Phosphatase	TCAGGGCAATGAGGTCACATC	CACAATGCCCACGGACTT
Bglap	Osteocalcin	GAGGACCATCTTTCTGCTCAC	CCAAGGTAGCGCCGGAGTCTG
Ccn1	Cellular Communication Network Factor 1	CTGCAGCAAAACTCAGCCCT	CACAGGGTCTGCCTTCTGAC
Ccnd1	Cyclin-D	GCGTACCCTGACACCAATCT	CTCTTCGCACTTCTGCTCCT
Ctnnb1	β-Catenin	GACTCACGCAGTGAAGAATG	GCTGTAGCAGGTTCACTAGA
Dkk1	Dickkopf-1 Wnt Signaling inhibitor	GCCTCCGATCATCAGACTGT	GCAGGTGTGGAGCCTAGAAG
Dmp1	Dentin Matrix Acid Phospho-protein 1	TGTCATTCTCCTTGTGTTCCTTTG	AGAGCTTTCAGATTCAGTATTGT GGTAT
E11	GP38 (podoplanin)	AAACGCAGACAACAGATAAGAAA GAT	GTTCTGTTTAGCTCTTTAGGGC GA
Hprt	Hypoxanthine Phosphoribosyl-transferase	CCAGACAAGTTTGTTGTTGGA	GCTTTGTATTTGGCTTTTCCA
Runx2	Runx-2	GACAGAAGCTTGATGACTCTAAA CC	TCTGTAATCTGACTCTGTCCTTG T
Sp7	Osterix	CGCTTTGTGCCTTTGAAAT	CCGTCAACGACGTTATGC
Wnt16	Wnt Family #16 Member 16	GGAGCTGTGCAAGAGGAAAC	AGTGGCGACCATACAGTTCC
