# Supplementary figures and images for: Protein kinase G2 activation restores Wnt signaling and bone mass in glucocorticoid-induced osteoporosis in mice
Source: JCI Insight. 2024 Jun 17;9(14):e175089. doi: 10.1172/jci.insight.175089 (PMC11383176; doi:10.1172/jci.insight.175089)

## Uncropped Western Blots for Fig. 7

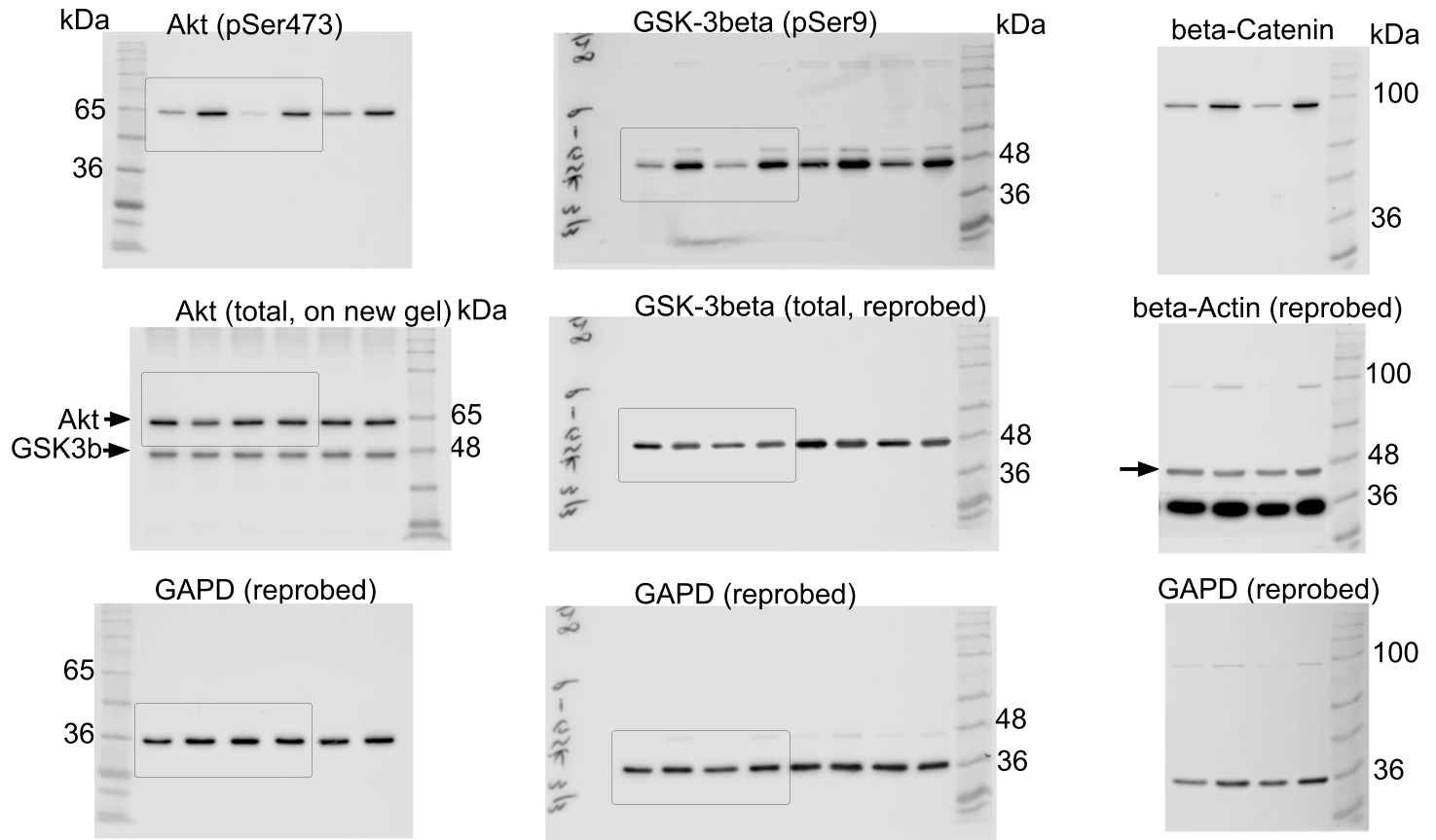

Supplement: Unedited blot and gel images [file jciinsight-9-175089-s087.pdf]
